# Comparative efficacy and safety of systemic therapies for moderate-to-severe atopic dermatitis in adults: a systematic review of randomized controlled trials

**DOI:** 10.3389/fimmu.2026.1985846

**Published:** 2026-10-30

**Authors:** Vasiliki Pikoula, Efterpi Zafiriou, Anthony Bewley, Andrea Carugno, Ignasi Figueras Nart, Emily Kelly, Nina Magnolo, Julia-Tatjana Maul, Marta Szepietowska, Jacek C. Szepietowski, Tiago Torres, Alexander Zink, Filomena Russo, Emanuele Scala, Maria-Angeliki Gkini

**Affiliations:** 1 Barts and The London School of Medicine and Dentistry, Queen Mary University of London, London, United Kingdom; 2 Queen Elizabeth Hospital King’s Lynn, King’s Lynn, United Kingdom; 3 Department of Dermatology, University Hospital of Larisa, University of Thessaly, Larisa, Greece; 4 Department of Dermatology, Barts Health NHS Trust, London, United Kingdom; 5 Dermatology Unit, Department of Medicine and Surgery, University of Insubria, Varese, Italy; 6 Department of Dermatology, Hospital Universitari de Bellvitge, L’Hospitalet de Llobregat, Barcelona, Spain; 7 University of Liverpool Medical School, Liverpool, United Kingdom; 8 Department of Dermatology, University Hospital of Muenster, Münster, Germany; 9 Department of Dermatology, University Hospital Zurich, Zurich, Switzerland; 10 Department of Dermatology, Jagiellonian University Medical College, Cracow, Poland; 11 Doctoral School of Medical and Health Sciences, Jagiellonian University Medical College, Cracow, Poland; 12 Division of Dermatology, Venereology and Clinical Immunology, Faculty of Medicine, Wroclaw University of Science and Technology, Wroclaw, Poland; 13 Department of Dermato-Venereology, 4th Military Hospital, Wroclaw, Poland; 14 Instituto de Ciências Biomédicas Abel Salazar, University of Porto, Porto, Portugal; 15 Department of Dermatology, Centro Hospitalar Universitário do Porto, Porto, Portugal; 16 Dermatology Clinic, Instituto Médico de Estudos Imunológicos, Porto, Portugal; 17 Department of Dermatology and Allergy, School of Medicine, Technical University of Munich, Munich, Germany; 18 Department of Dermatology, Istituto Dermopatico dell’Immacolata, Istituto Dermopatico dell'Immacolata-Istituto di Ricovero e Cura a Carattere Scientifico (IDI-IRCCS), Rome, Italy; 19 Experimental Immunology Lab, Istituto Dermopatico dell’Immacolata, Istituto Dermopatico dell'Immacolata-Istituto di Ricovero e Cura a Carattere Scientifico (IDI-IRCCS), Rome, Italy

**Keywords:** atopic dermatitis, biologic therapy, dupilumab, EASI-75, eczema, JAK inhibitors, randomized controlled trials, systematic review

## Abstract

**Background:**

Atopic dermatitis (AD) is a common, chronic inflammatory skin condition, and patients with moderate-to-severe disease often require systemic therapy. Since 2017, targeted systemic agents, biologics and Janus kinase (JAK) inhibitors, have expanded treatment options available, but comparative evidence remains limited by heterogeneous trial design, outcome reporting, and follow-up duration.

**Objective:**

To systematically review the efficacy and safety of European Medicines Agency (EMA)-approved systemic therapies for moderate-to-severe AD in adults.

**Methods:**

Following PRISMA 2020 guidelines, we searched PubMed/MEDLINE, Embase and Web of Science for randomized controlled trials evaluating EMA-approved biologics (dupilumab, tralokinumab, lebrikizumab, nemolizumab) and JAK inhibitors (upadacitinib, abrocitinib, baricitinib) at recommended adult doses. The primary outcome was the proportion of patients achieving EASI-75 at the primary trial endpoint (12–16 weeks). Due to substantial clinical and methodological heterogeneity, a structured narrative synthesis was conducted rather than a meta-analysis.

**Results:**

Twenty-five RCTs met inclusion criteria. At EMA-recommended doses, EASI-75 responses ranged from 44% to 69% for dupilumab, 60.6% for lebrikizumab, 25-64% for tralokinumab, and 49.1% for nemolizumab (combination-therapy data only). Among JAK inhibitors, upadacitinib showed the highest observed response rate (73.0% at 16 weeks), followed by abrocitinib (64.6% at 12 weeks) and baricitinib (17.9-48.0%). Placebo response rates ranged from 6% to 36% and were consistently higher in combination-therapy trials. Any adverse event occurred in 45.2% (abrocitinib 100 mg) to 82.46% (nemolizumab) of patients, with serious adverse events generally low across therapies.

**Conclusion:**

EMA-approved systemic therapies for moderate-to-severe AD demonstrate meaningful short-term efficacy and acceptable safety. However, treatment selection should remain individualized given cross-trial heterogeneity and the current absence of long-term comparative data. Further head-to-head trials and real-world evidence are needed to optimize treatment sequencing.

**Systematic Review Registration:**

https://www.crd.york.ac.uk/prospero/, identifier CRD420251102911

## 1 Introduction

Atopic dermatitis (AD) is the most common chronic inflammatory skin condition, affecting up to 15-20% of children and as many as 10% of adults worldwide, with a rising prevalence in developed countries (1, 2). It is characterized by recurrent flare-ups, severe itching, and skin barrier dysfunction, significantly impacting quality of life, including sleep disturbances, psychological stress, and poor school performance in children, as well as reduced work productivity in adults (3, 4). Disease severity is usually evaluated using validated tools such as the Eczema Area and Severity Index (EASI), the Investigator’s Global Assessment (IGA), or SCORing Atopic Dermatitis (SCORAD) (5, 6).

Mild disease is typically treated with topical agents, including emollients, topical corticosteroids, and topical calcineurin inhibitors. However, patients with more extensive or resistant disease often require systemic therapy (7, 8). Until 2017, systemic treatments for AD were limited to traditional immunosuppressants like ciclosporin, azathioprine, and methotrexate, which have been linked to significant side effects and lack specificity in targeting disease mechanisms (9, 10).

Atopic dermatitis is primarily driven by a type 2 inflammatory response, associated with elevated levels of key cytokines, including interleukin (IL)-4, IL-13, and IL-31 (1). Advances in understanding these immunological mechanisms have led to the development of targeted systemic therapies that selectively inhibit these pathways (1). Monoclonal antibodies, such as dupilumab, block IL-4 receptor alpha, thereby inhibiting IL-4/IL-13 signaling, while tralokinumab and lebrikizumab block IL-13 (10). Conversely, Janus kinase (JAK) inhibitors, including upadacitinib, abrocitinib, and baricitinib, target broader intracellular signaling pathways involved in multiple cytokine cascades (11, 12). Most recently, the European Commission approved nemolizumab, an anti-IL-31 receptor A antibody, for moderate-to-severe AD on 12 February 2025, following a positive opinion from the EMA, further expanding the range of systemic biologic options (13, 26). This approval date, confirmed via the EMA’s public assessment report, precedes this review’s eligibility cutoff of 23 February 2025. EMA approval dates for the included therapies range from 2017 (dupilumab) to February 2025 (nemolizumab) (26).

Despite these advances, key questions remain regarding the comparative efficacy and safety of these agents. Existing trials vary in design, outcome measures, and duration, which limits direct comparisons across therapies. A living systematic review and network meta-analyses, most recently updated in 2024, has synthesized RCT data on systemic therapies using continuous outcomes (e.g., EASI, DLQI), but often excludes nemolizumab, which was addressed separately in a subsequent 2025 update (14). A recent network meta-analysis has also assessed pruritus outcomes using the Numerical Rating Scale (NRS), further emphasizing the heterogeneity in outcome reporting across studies (15).

A therapy was considered EMA-approved if authorization for moderate-to-severe AD had been granted as of 23 February 2025. Conventional systemic immunosuppressants (e.g. ciclosporin, methotrexate, azathioprine) were excluded, as they lack an AD-specific EMA-approved indication and are typically used off-label with heterogeneous dosing regimens, limiting comparability with mechanism-targeted agents. This review focuses on induction-phase efficacy and safety at the primary trial endpoint (12–16 weeks), with maintenance and long-term outcomes outside the review’s scope.

This systematic review aims to address these limitations by combining data from randomized controlled trials (RCTs) evaluating EMA-approved systemic therapies at their recommended doses in adults with moderate-to-severe atopic dermatitis. The main goal is to compare clinically meaningful efficacy outcomes, focusing on EASI 75 response rates assessed at the primary trial endpoint (usually 12–16 weeks). Secondary outcomes include higher-threshold efficacy measures (EASI-90 and IGA0/1, which was co-primary alongside EASI-75 in most included trials), safety, and treatment discontinuation rates. Additional outcomes, such as SCORAD and patient-reported measures like DLQI, are summarized narratively due to differences in reporting. By including recently approved agents and classifying results by drug class and trial features, this review seeks to provide a detailed overview of current evidence to support clinical decision-making in managing moderate-to-severe atopic dermatitis.

## 2 Methods

### 2.1 Study design and protocol

This systematic review followed PRISMA 2020 guidelines. A study protocol was created before starting to define objectives, eligibility criteria, data extraction methods, and quality assessment approach. The main goal was to evaluate the effectiveness and safety of EMA-approved targeted systemic therapies, including biologic agents (anti-Th2 cytokine therapies) and Janus kinase (JAK) inhibitors, for treating moderate-to-severe atopic dermatitis (AD) in adults. We restricted inclusion to individual randomized controlled trials (RCTs) that represent Level 2 evidence according to the Oxford Centre for Evidence-Based Medicine (CEBM) 2011 hierarchy (16), given the methodological strengths of RCT design in minimizing bias and confounding (17).

Registration: Protocol registered in PROSPERO (CRD420251102911) on 12 July 2025. Available at: https://www.crd.york.ac.uk/PROSPERO/display_record.php?ID=CRD420251102911.

### 2.2 Eligibility

#### 2.2.1 Inclusion criteria

Studies were selected according to the following eligibility criteria:

- Study design: RCTs published as full-text, peer-reviewed articles in English.
- Population: Adults (≥18 years) with moderate-to-severe AD defined using validated criteria, including Investigator’s Global Assessment (IGA) ≥3, Eczema Area and Severity Index (EASI) ≥16, or equivalent severity thresholds as specified in individual trials.
- Interventions: EMA-approved systemic therapies, including biologics (dupilumab, tralokinumab, lebrikizumab, nemolizumab) and Janus kinase (JAK) inhibitors (upadacitinib, abrocitinib, baricitinib). Both monotherapy and combination therapy with topical anti-inflammatory treatments were eligible.
- Comparators: placebo, standard therapy, or an active comparator (another systemic agent).
- Outcomes:

- At least one validated efficacy outcome evaluated at the primary endpoint (usually 12–16 weeks), including:
  - Primary outcome:
    ▪ EASI 75 (at least 75% improvement from baseline).
  - Secondary outcomes:
    ▪ EASI-90, where reported
    ▪ IGA 0/1, with or without ≥2-point improvement from baseline (as defined by individual trials)
    ▪ Additional outcomes, including SCORAD and patient-reported measures such as the Dermatology Life Quality Index (DLQI), where available.
    ▪ At least one safety outcome (e.g., overall adverse events, serious adverse events, infections, malignancies, cardiovascular events).

Therapies approved before the eligibility cutoff were included regardless of approval date relative to the literature search dates, provided qualifying trial data were identified within the search period.

#### 2.2.2 Exclusion criteria

- Non-randomized studies, case series/reports, conference abstracts, editorials, or reviews.
- Pediatric-only studies that do not include separate adult data.
- Trials of non-systemic treatments (e.g., topical agents, phototherapy) or unapproved investigational agents.
- Studies lacking relevant efficacy or safety outcomes.

A detailed summary of the eligibility criteria is available in **Supplementary Table S1**.

A list of potentially eligible mixed-age trials excluded under this criterion is provided in **Supplementary Table S3**.

### 2.3 Search strategy

A systematic literature search was performed in PubMed/MEDLINE, Embase, and Web of Science using a combination of controlled vocabulary (e.g., MeSH terms) and free-text terms with Boolean operators. Core search concepts included AD/eczema, EMA-approved targeted systemic agents (dupilumab, tralokinumab, lebrikizumab, nemolizumab, upadacitinib, abrocitinib, baricitinib), and efficacy and safety outcomes. Search strategies were tailored for each database. Full search details, including search terms, limits, and dates, are available in **Supplementary Material 1**.

### 2.4 Study selection

Search results were imported into Covidence for duplicate removal and screening. Two reviewers independently screened titles and abstracts, followed by full-text assessment against predefined eligibility criteria. Discrepancies were resolved through discussion and consensus, involving a third reviewer if needed.

The study selection process is shown in the PRISMA flow diagram (Results).

### 2.5 Data extraction

Two reviewers independently recorded data in a pilot-tested, standardized spreadsheet. The data extracted included the following:

- Study characteristics: first author, journal, year of publication, geographic location, number of study sites, and trial duration.
- Participant characteristics: sample size, age, sex, baseline disease severity, relevant comorbidities (including atopic comorbidities such as asthma), where reported.
- Interventions: drug, dosage regimen, route of administration, treatment duration, use of rescue therapy.
- Efficacy outcomes: EASI 75, EASI-90, IGA 0/1, and/or SCORAD.
- Safety outcomes: rates of overall and serious adverse events; infections (e.g., URTI, HSV, VZV), conjunctivitis, gastrointestinal or cardiovascular events, venous thromboembolism (VTE), malignancy, laboratory abnormalities, and other treatment-emergent adverse events as reported in individual trials.
- Adherence/discontinuation: dropout rates and reasons for discontinuation.
- Statistical methods: handling for missing data (e.g., non-responder imputation (NRI)), analysis populations, and any prespecified subgroup analyses.

### 2.6 Risk of bias

Risk of bias for each included RCT was evaluated using the Cochrane Risk of Bias 2 (RoB 2) tool across five domains: (1) randomization process, (2) deviations from intended interventions, (3) missing outcome data, (4) measurement of the outcome, (5) and selection of the reported result (18). Two reviewers assessed studies independently and any discrepancies were resolved through consensus.

### 2.7 Data synthesis

Due to substantial clinical and methodological heterogeneity across included studies, including differences in study design, patient populations, dosing regimens, background therapies, outcome definitions, and timepoint of assessment, a meta-analysis was not performed. This judgement was made on clinical and methodological grounds rather than a statistical heterogeneity test, given the absence of pooled estimates. Key drivers included variable background corticosteroid use, differing placebo response between monotherapy and combination designs, and inconsistent handling of missing data across trials. Instead, a structured narrative synthesis was conducted.

Analyses focused on EMA-recommended adult dosing regimens and were stratified by treatment approach (monotherapy vs combination therapy with background topical corticosteroids and/or topical calcineurin inhibitors). Head-to-head active-comparator trials were analyzed separately from placebo-controlled trials, and indirect quantitative comparisons between treatments were not performed.

Non-EMA dosing regimens and dose-response findings were summarized descriptively. DLQI and SCORAD outcomes were also summarized descriptively due to heterogeneous and incomplete reporting across studies.

## 3 Results

### 3.1 Systematic review

The database search yielded 830 records. After deduplication and screening, 25 randomized controlled trials (RCTs) met the inclusion criteria and were included in the review. The study selection process is shown in **Figure 1** (PRISMA) (19).

**Figure 1:**
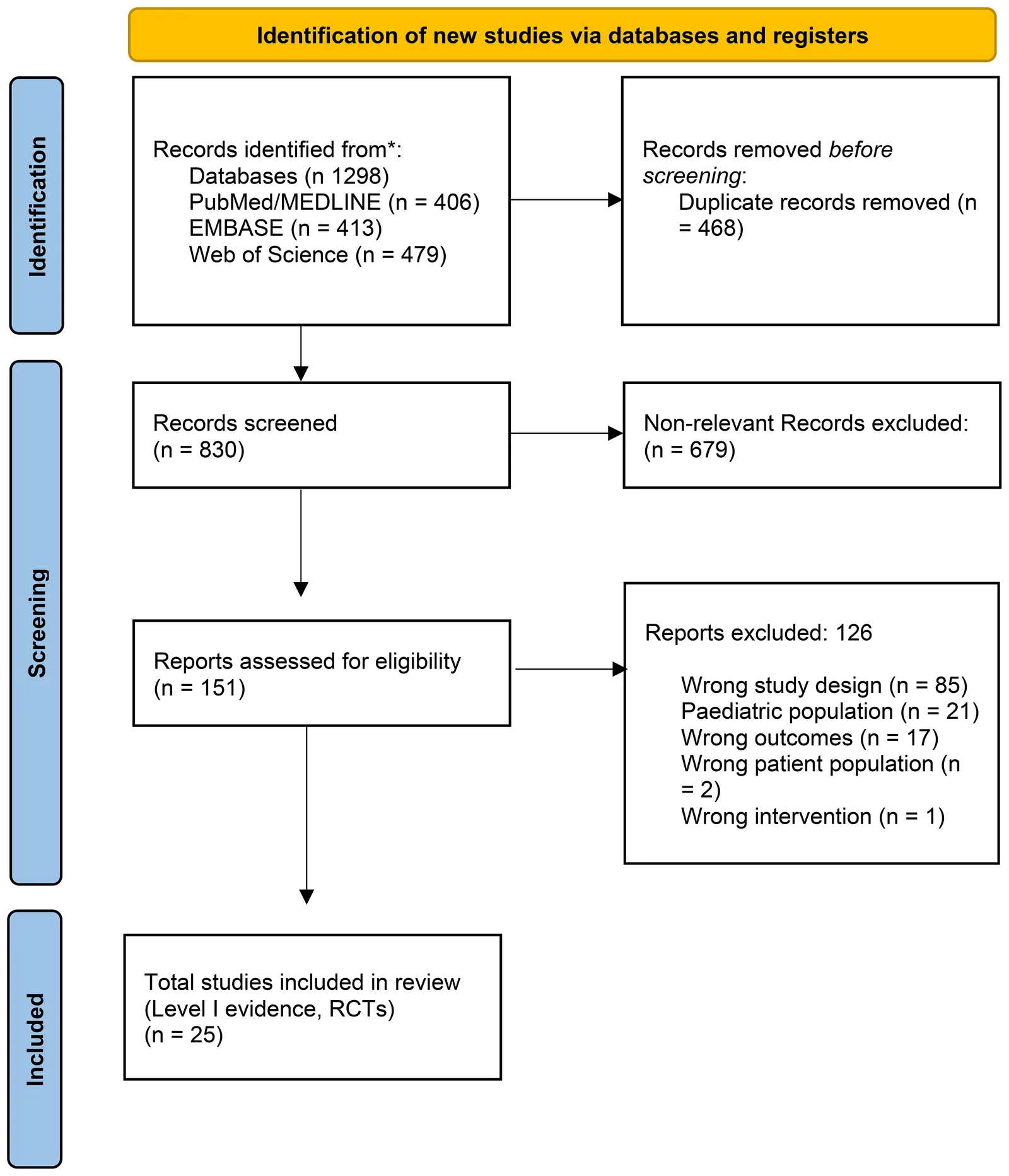
PRISMA study attrition diagram, showcasing the process of study selection.

### 3.2 Study characteristics

The 25 RCTs were multicenter and double-blind, of which 22 were placebo-controlled and 3 used an active comparator (dupilumab, in JADE COMPARE, Heads Up, JADE DARE). Thirteen trials evaluated monotherapy and 12 evaluated combination therapy with background topical corticosteroids. All 25 enrolled adults with moderate-to-severe atopic dermatitis (AD) and evaluated seven EMA-approved systemic therapies: four biologics (dupilumab, tralokinumab, lebrikizumab, nemolizumab) and three JAK inhibitors (upadacitinib, abrocitinib, baricitinib).

Most trials reported 16-week outcomes. However, four used 12-week primary assessments (DUPISTAD, TREBLE, Ruzicka et al., 2017, and Gooderham et al., 2019). Baseline characteristics were broadly comparable across studies (**Supplementary Table S4**).

### 3.3 Risk of bias

Using Cochrane RoB 2, most studies were rated as having some concerns of bias across domains. “Some concerns’ mostly stemmed from missing outcome data and reliance on non-responder imputation (NRI) without sensitivity analyses (**Supplementary Table S15**). Risk-of-bias patterns were broadly similar between biologic and JAK inhibitor trials, with “Some concerns” driven predominantly by missing outcome data in both classes with no domain showing a class-specific pattern of bias. No trial received an overall high-risk rating, supporting the internal validity of the individual trial-level efficacy and safety estimates. This does not extend to the validity of descriptive comparisons drawn across trials, which are addressed separately as a limitation of this review.

### 3.4 Efficacy

#### 3.4.1 Primary outcome: EASI 75 at EMA-recommended doses

All 25 trials reported the proportion of patients achieving EASI 75 (at least 75% improvement from baseline). EASI 75 was chosen as the primary trial endpoint (at 12 or 16 weeks) because it is a clinically meaningful and consistently reported measure across trials. Efficacy results are shown for EMA-recommended dosing regimens, using study-level estimates reported without pooling (**Tables 1**, **2**; **Supplementary Table S5**).

**Table 1:** EASI 75 response rates at EMA-recommended doses of biologics (dupilumab, tralokinumab, nemolizumab, lebrikizumab) across included randomized controlled trials.

Study label	Dose	Week	EASI 75 (%)	Mono/Combo
Dupilumab				
Zhao et al., 2022	300 mg Q2W + 600 mg LD	16	57.3%	Mono
LIBERTY AD CHRONOS	300 mg Q2W	16	69.0%	Combo
DUPISTAD	300 mg Q2W + LD	12	60.6%	Combo
SOLO 1	300 mg Q2W + 600 mg LD	16	51.0%	Mono
SOLO 2	300mg Q2W + 600 mg LD	16	44.0%	Mono
LIBERTY AD CAFÉ	300 mg Q2W + 600 mg LD	16	62.6%	Combo
Tralokinumab				
ECZTRA 1	300 mg Q2W + 600 mg LD	16	25.0%	Mono
ECZTRA 2	300 mg Q2W + 600 mg LD	16	33.2%	Mono
ECZTRA 3	300 mg Q2W + 600 mg LD	16	56.0%	Combo
ECZTRA 7	300mg Q2W + 600 mg LD	16	64.2%	Combo
Nemolizumab				
Silverberg_2020_JACI	30 mg Q4W + 60 mg LD	16	49.1%	Combo
Lebrikizumab				
Guttman-Yassky et al., 2020	250 mg Q2W + 2 × 500 mg LD	16	60.6%	Mono

Study-level proportions achieving EASI 75 (≥75% improvement from baseline) are reported at the primary efficacy time point (Week 12 or 16). Values are reported as percentages (%). Head-to-head trials excluded. See **Supplementary Table S5** for study-level data and denominators.

**Table 2:** EASI 75 response rates at EMA-recommended doses of JAK inhibitors (abrocitinib, upadacitinib, baricitinib) across included randomized controlled trials.

Study label	Dose	Week	EASI 75 (%)	Mono/Combo
Abrocitinib				
Gooderham et al., 2019	100mg once daily	12	40.7%	Mono
Gooderham et al., 2019	200mg once daily	12	64.6%	Mono
Upadacitinib				
Guttman-Yassky et al., 2020	15 mg once daily	16	59.0%	Mono
Guttman-Yassky et al., 2020	30 mg once daily	16	73.0%	Mono
Baricitinib				
BREEZE-AD1	2 mg once daily	16	18.7%	Mono
BREEZE-AD1	4 mg once daily	16	24.8%	Mono
BREEZE-AD2	2 mg once daily	16	17.9%	Mono
BREEZE-AD2	4 mg once daily	16	21.1%	Mono
BREEZE-AD4	2 mg once daily	16	30.0%	Combo
BREEZE-AD4	4mg once daily	16	37.0%	Combo
BREEZE-AD7	2 mg QD	16	43.0%	Combo
BREEZE-AD7	4 mg QD	16	48.0%	Combo
Guttman-Yassky et al., 2019	2 mg once daily	16	30.0%	Combo
Guttman-Yassky et al., 2019	4 mg once daily	16	34.0%	Combo

Study-level proportions achieving EASI 75 (≥75% improvement from baseline) are reported at the primary efficacy time point (Week 12 or 16). Values are reported as percentages (%). Head-to-head trials excluded. See **Supplementary Table S5** for study-level data and denominators.

##### 3.4.1.1 Key findings

Across therapies, EASI 75 responses at 12–16 weeks varied between agents and remained consistent within drug classes (**Tables 1**, **2**).

Among biologics, dupilumab (300 mg Q2W) consistently showed high efficacy, with EASI 75 responses ranging from 44% to 69% across trials. Higher response rates were observed in studies that followed background topical therapy. Lebrikizumab (250 mg Q2W) reached an EASI 75 response of 60.6% at 16 weeks in monotherapy trials, while tralokinumab (300 mg Q2W) showed more variable responses (25-64% at 16 weeks), with better efficacy in trials allowing background topical corticosteroids (TCS). Notably, all nemolizumab efficacy data at the EMA-recommended dose came from a single combination-therapy trial, precluding comparison of its monotherapy efficacy. Nemolizumab (30 mg Q4W) reported an EASI 75 of 49.1% at 16 weeks in combination therapy settings.

The highest observed EASI 75 response among JAK inhibitors monotherapy trials was Upadacitinib (30 mg OD, 73.0% at 16 weeks), followed by abrocitinib (200 mg once daily) with 64.6% at 12 weeks. Baricitinib trials reported more moderate efficacy across trials (17.9-48.0% at 16 weeks), with variability influenced by trial design and background therapy.

Placebo EASI 75 responses ranged from 6% to 36% and were consistently higher in combination-therapy studies than in monotherapy trials, reflecting the influence of background topical therapy and study setting on the interpretation of treatment efficacy.

##### 3.4.1.2 Monotherapy versus combination therapy

Combination-therapy trials that allowed background TCS showed higher EASI 75 response rates for both active treatments and placebo compared to monotherapy trials. This trend was most noticeable for dupilumab and tralokinumab, while lebrikizumab and JAK inhibitors showed less pronounced differences (**Tables 1**–**3**).

**Table 3:** Descriptive summary of EASI 75 response rates at EMA-recommended doses, stratified by treatment regimen.

Dose	Week	EASI 75 (%)	Mono/Combo
Dupilumab			
300 mg Q2W + 600 mg LD	16	50.8%	Mono
	16	64.1%	Combo
Tralokinumab			
300 mg Q2W + 600 mg LD	16	29.1%	Mono
	16	60.1%	Combo
Nemolizumab			
30 mg Q4W + 60 mg LD	16	49.1%	Combo
Lebrikizumab			
250 mg Q2W + 2 × 500 mg LD	16	60.6%	Mono
Abrocitinib			
100mg once daily	12	40.7%	Mono
200mg once daily	12	64.6%	Mono
Upadacitinib			
15 mg once daily	16	59.0%	Mono
30 mg once daily	16	73.0%	Mono
Baricitinib			
2 mg once daily	16	18.3%	Mono
	16	34.3%	Combo
4 mg once daily	16	22.9%	Mono
	16	39.7%	Combo

Values are reported as percentages and represent unweighted arithmetic means of study-level response rates from trials using comparable dosing regimens. No sample-size weighting, formal pooling, or comparative statistical analysis was performed.

##### 3.4.1.3 Active comparator (head-to-head) trials

JADE COMPARE (abrocitinib 200 mg vs dupilumab 300 mg) and Heads Up (upadacitinib 30 mg vs dupilumab 300 mg) showed higher EASI 75 results with the JAK inhibitors (71.0% vs 65.5% and 72.4% vs 62.6%). JADE DARE (with background TCS) showed a similar pattern (77.3% vs. 67.8%, **Figure 2**), though EASI 75 was a secondary rather than a prespecified primary or key secondary endpoint in this trial. Heads Up was the only trial in which EASI 75 was a formally powered primary comparison. These data suggest that JAK inhibitors were associated with higher short-term response rates in these specific head-to-head comparisons though findings should not be extrapolated to comparisons involving other agents not directly studied against dupilumab.

**Figure 2:**
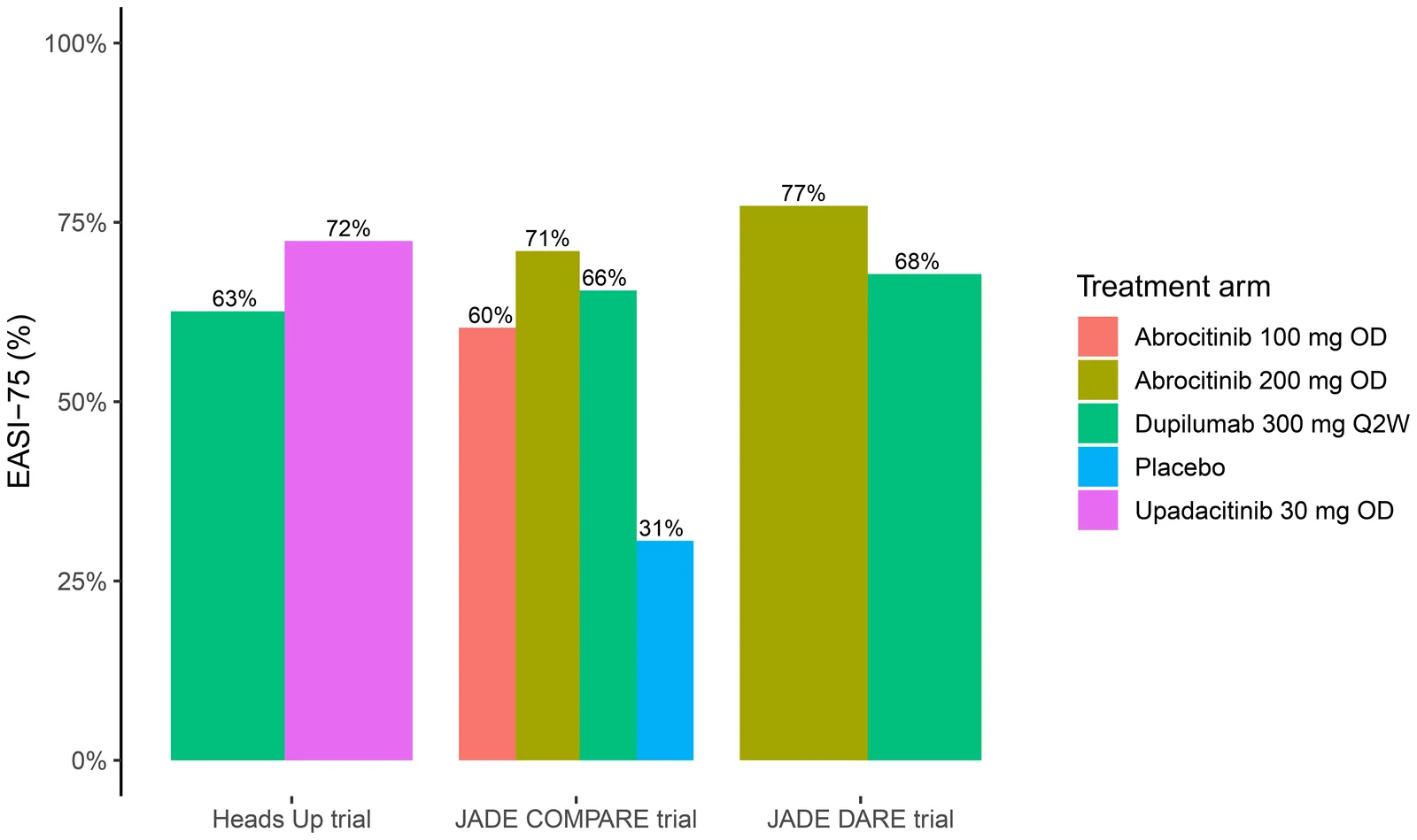
Head-to-head EASI 75 response rates from selected randomized controlled trials. EASI 75 response rates (%) at 16 weeks are shown for the Heads Up trial, JADE COMPARE trial, and JADE DARE trial. Values represent the proportion of patients achieving at least 75% improvement from baseline in the Eczema Area and Severity Index (EASI 75). Data are presented for individual treatment groups without pooling.

#### 3.4.2 Key secondary outcomes

##### 3.4.2.1 IGA 0/1

At EMA-recommended doses, IGA 0/1 responses ranged from 15.8% to 44.6% at the primary trial endpoint (12 or 16 weeks), with variation observed across different drug classes and treatment settings.

Among JAK inhibitors, the highest observed IGA 0/1 response was with Upadacitinib 30 mg (50.0%), with abrocitinib 200 mg reaching 43.8%, both in monotherapy settings. Baricitinib trials reported lower responses overall (10.6%-31.0%).

Among biologics, the highest observed IGA 0/1 response was with lebrikizumab (250mg Q2W, 44.6%), with dupilumab trials reporting dupilumab 26.8-40.2%. Tralokinumab responses varied (15.8-38.9%), and nemolizumab (30mg Q4W) reached 33.3%. Placebo responses ranged from 4.5% to 26.2%. As all included trials required IGA of 3 or 4, achieving IGA 0/1 necessarily reflects at least a 2-point improvement in nearly all cases.

Among active-comparator (head-to-head) trials, abrocitinib 200 mg showed a higher IGA 0/1 response than dupilumab, at 47.5% vs 38.8% in JADE COMPARE, and 55.3% versus 42.5% in JADE DARE. IGA 0/1 was not reported for the Heads Up trial.

IGA 0/1 response rates are summarized in **Supplementary Tables S8** and **S9**.

##### 3.4.2.2 EASI-90

EASI-90 response rates at EMA-recommended doses, excluding head-to-head trials, ranged from 8.9% to 52.1%, with variation across different drug classes and treatment settings.

Among JAK inhibitors, the highest observed EASI-90 was with abrocitinib 200mg (52.1% at 12 weeks) in monotherapy, followed by upadacitinib 30 mg (50.0% at 16 weeks), also in monotherapy. Baricitinib trials had the lowest efficacy overall (8.9-25.0% at 16 weeks), with higher response rates seen in combination therapy trials compared to monotherapy.

Among biologics, the highest observed EASI-90 responses among biologics with lebrikizumab (44.0% of monotherapy trials), and dupilumab (30.0% to 45.8%), with higher response rates seen in combination therapy trials compared to monotherapy. Tralokinumab exhibited more variable responses (14.5–41.1%), with higher values in combination trials. Nemolizumab trials observed responses of 33.3% in combination therapy trials.

Placebo EASI-90 response rates ranged from 0% to 29.3%, with higher responses again observed in combination-therapy trials.

Among the head-to-head trials, JAK inhibitors again showed numerically higher EASI-90 responses than dupilumab. In the Heads Up trial, upadacitinib achieved a 61.6% response compared to 40.3% for dupilumab. In the JADE COMPARE and JADE DARE trials, abrocitinib 200 mg achieved responses of 48.9% and 54.0% compared with 38.8% to 42.0% for dupilumab.

EASI-90 response rates are summarized in **Supplementary Tables S6** and **S7**.

#### 3.4.3 Dose–response and non-recommended regimens

Clear dose-response effects were observed for JAK inhibitors, especially upadacitinib (EASI 75: 24.0% at 7.5 mg, 59.0% at 15 mg, 73.0% at 30 mg) and abrocitinib (13–17% at 10–30 mg, 40.7% at 100 mg, 64.6–77.3% at 200 mg). In contrast, biologics showed relatively consistent efficacy across different dosages. Nemolizumab exhibited a non-linear dose-response, with EASI 75 rates of 32.3% (0.1 mg/kg), 37.7% (0.5 mg/kg), and 22.3% (2.0 mg/kg), and 49.1% at the EMA-approved 30 mg Q4W, suggesting higher effectiveness at intermediate doses.

#### 3.4.4 Patient-reported and composite outcomes (narrative)

##### 3.4.4.1 SCORAD

SCORAD outcomes were reported inconsistently and are presented descriptively. Active treatments consistently yielded greater reductions than placebo, with mean absolute reductions of 25.2 to 42.7 points for biologics, and mean percentage reductions of 47.0% to 69.7%, for dupilumab, baricitinib, and abrocitinib. Where reported, SCORAD -50/75 responses were higher with active therapy, reaching 34.6–66.4% for dupilumab and lebrikizumab, 6–18% for baricitinib, and 16.2% (SCORAD-75) for nemolizumab, versus 1–26% with placebo. SCORAD response rates are summarized in **Supplementary Table S10**.

##### 3.4.4.2 DLQI

Where reported, active treatments showed greater improvements in Dermatology Life Quality Index (DLQI) scores compared to placebo, with mean reductions of approximately 4.3–11.8 points at the primary assessment timepoint (12–16 weeks). DLQI ≥4-point responder rates reached 72.2-87.6% for dupilumab, 44.6-83.5% for tralokinumab, 74.7–86.4% for abrocitinib, and 17–23% for baricitinib. Placebo DLQI response rates ranged from 7% to 65.9% across trials.

DLQI outcomes differed between studies and reporting formats and are summarized in **Supplementary Table S11**.

### 3.5 Safety

A summary of adverse events (AEs) is provided in **Table 4** with study-level data for all the tested doses in **Supplementary Table S12**. The proportion of patients experiencing any adverse event ranged from 45.2% (abrocitinib 100 mg) to 82.46% (nemolizumab). Serious AEs were generally infrequent, mostly between 1.6% and 3.7% across doses, with the highest rates seen with baricitinib 4 mg (3.7%) and nemolizumab (3.51%). Given the exclusion of pivotal mixed-age trials under this review’s adult-only criterion (**Supplementary Table S3**), the safety populations for nemolizumab (N = 57) and Upadacitinib (N = 426) are substantially smaller than their full phase 3 safety databases, limiting the ability to detect infrequent adverse events for these agents relative to more established therapies such as dupilumab (N = 1829).

**Table 4:** Safety overview for included therapies at EMA-recommended doses, presented separately by dose where more than one dose is approved.

Drug	N(total)	Any AE n (%)	Serious AE n (%)	D/C due to AE n (%)	Most common AE, n/N (%) [studies]
Abrocitinib 200 mg QD	643	416 (64.7)	10 (1.6)	23 (3.6)	Nausea, 95/588 (16.2) [2/3]
Abrocitinib 100 mg QD	294	133 (45.2)	9 (3.1)	9 (3.1)	Headache, 15/294 (5.1) [2/2]
Upadacitinib 30 mg QD	384	282 (73.4)	10 (2.6)	11 (2.9)	Acne, 61/384 (15.9) [2/2]
Upadacitinib 15 mg QD	42	32 (76.2)	1 (2.4)	2 (4.8)	URTI, 5/42 (11.9) [1/1]
Baricitinib 4 mg QD	489	312 (63.8)	18 (3.7)	16 (3.3)	Nasopharyngitis, 73/451 (16.2) [4/5]
Baricitinib 2 mg QD	577	369 (64.0)	14 (2.4)	15 (2.6)	Nasopharyngitis, 78/540 (14.4) [4/5]
Dupilumab 300 mg Q2W	1829	1205 (65.88%)	32 (1.75%)	36 (1.97%)	Conjunctivitis, 125/1172 (10.7) [5/9]
Lebrikizumab 250 mg Q2W	75	46 (61.33%)	2 (2.67%)	3 (4.00%)	Nasopharyngitis, 9/75 (12.0) [1/1]
Tralokinumab 300 mg Q2W	1582	1111 (70.23%)	36 (2.28%)	36 (2.28%)	Viral URTI, 274/1582 (17.3) [4/4]
Nemolizumab 30 mg Q4W	57	47 (82.46%)	2 (3.51%)	2 (3.51%)	Nasopharyngitis and AD (tied), 14/57 (24.6) each [1/1]

N(total) represents the sum of all randomized patients in active-treatment arms only (placebo arms excluded) receiving the specified EMA-recommended dose, across included trials. The most common AE is reported as pooled n/N across only the trials listed that event among their top three most-reported adverse events for that dose, with [x/y] indicating how many of the y trials at that dose reported it. Study-level data for tested doses, including non-EMA-recommended, are provided in **Supplementary Table S12**.

#### 3.5.1 Discontinuations

Discontinuation rates include all withdrawals occurring up to the primary efficacy timepoint (week 12 or 16). Discontinuations due to adverse events ranged across trials, generally occurring in less than 5% of patients. Rates ranged from 1.97% with dupilumab to 4.8% with upadacitinib 15 mg, the latter based on a small arm (N = 42). Among JAK inhibitors, rates ranged from 2.6% with baricitinib 2 mg to 4.8% with upadacitinib 15 mg. Across studies, discontinuations were more attributed to withdrawal of consent or lack of efficacy, particularly in placebo arms. No consistent pattern of treatment-limiting toxicity was observed across drug classes. Detailed reasons are summarized in **Supplementary Tables S13** and **S14**.

#### 3.5.2 Biologics

Among biologic therapies, conjunctivitis and upper respiratory tract infections were the most reported adverse events. Conjunctivitis was reported more frequently with dupilumab (6.2% to 28.0%) compared to tralokinumab (3.0% to 11.1%) across trials. Tralokinumab more frequently caused viral upper respiratory tract infections (8.3% to 26.8%) and atopic dermatitis flares (16.6% to 25.9%). Lebrikizumab was associated with upper respiratory tract infections or nasopharyngitis (8.2% to 12.0%), while conjunctivitis and injection site reactions occurred less often. Nemolizumab was most associated with nasopharyngitis (10.0% to 24.6%) and atopic dermatitis exacerbations or flares (19.0% to 24.6%). Additional adverse events, such as asthma-related events and peripheral edema, were reported in some studies but were not consistently observed across all trials. Injection site reactions were also noted in various biologic trials, especially with dupilumab and lebrikizumab, with rates reaching 8–19% in dupilumab studies and 9.3% with lebrikizumab 250 mg every two weeks.

#### 3.5.3 JAK inhibitors

In contrast, JAK inhibitors showed a wider range of systemic and dose-dependent side effects. Abrocitinib was most linked to nausea (4.2% to 19.0%), with the highest rates at 200 mg, as well as headache (4.2% to 13.0%) and acne/folliculitis (2.9% to 13.0%). Upadacitinib was most associated with acne (4.8% to 16.1%), and reports also included headache and creatinine phosphokinase (CPK) elevations. Baricitinib most often caused nasopharyngitis (8.1% to 37.0%) and CPK elevations (3.0% to 29.3%), especially in combination therapy trials, with headaches also noted in several studies (5.0% to 13.0%).

#### 3.5.4 Serious adverse events and malignancy

Serious adverse event rates were low across all therapies (**Table 4**), with no consistent differences observed between drug classes. No venous thromboembolism (VTE) events were reported in the included RCTs. There were isolated malignancy events (e.g., breast cancer, non-melanoma skin cancer, melanoma) reported in both active and placebo groups, but no consistent drug-specific safety signal was identified. Given the short trial durations and limited sample sizes for individual agents, this review cannot exclude clinically meaningful differences in rare or long-latency events such as malignancy or venous thromboembolism.

## 4 Discussion

This systematic review synthesized evidence from 25 RCTs evaluating EMA-approved systemic therapies for adults with moderate-to-severe atopic dermatitis (AD). Across studies, all EMA-approved systemic therapies were associated with clinically meaningful improvements in disease severity compared with placebo at 12–16 weeks, supporting their role as key treatment options in moderate-to-severe AD.

When evaluated using EMA-recommended dosing schedules, distinct efficacy patterns were evident within and across drug classes. Among biologics, dupilumab consistently showed robust EASI 75 responses in both monotherapy and combination therapy trials consistent with its established role in current treatment guidelines. Lebrikizumab also reported high response rates in monotherapy, while tralokinumab showed more variability, with higher efficacy observed in trials allowing background topical corticosteroids (TCS). Nemolizumab demonstrated meaningful efficacy in combination therapy, although evidence remains limited due to fewer trials and its more recent approval. Among JAK inhibitors, higher-dose regimens of upadacitinib and abrocitinib were associated with particularly high short-term efficacy, with EASI 75 response rates exceeding 70% in some trials.

These findings, along with consistent dose-response relationships, are consistent with a potential role of JAK where rapid disease control is a clinical priority, though this interpretation is based on descriptive, non-adjusted comparisons and would require confirmation through head-to-head evidence. In contrast, baricitinib trials reported more modest EASI 75 responses overall, with better responses observed in combination therapy approaches, highlighting the role of background treatment and study context.

Differences between monotherapy and combination-therapy trials should be interpreted with caution, as improved outcomes in studies allowing background topical therapy may partly reflect additive effects rather than the inherent efficacy of the systemic agent alone. These findings underscore the importance of interpreting efficacy data in the context of study design rather than attributing differences solely to the systemic intervention.

Evidence from three head-to-head randomized trials (JADE COMPARE, Heads Up, and JADE DARE) offers important comparative insights. These studies suggest that JAK inhibitors have greater short-term efficacy than dupilumab at 16 weeks, with one trial indicating a statistically significant advantage for abrocitinib in a combination-therapy setting. This supports a potential role for JAK inhibitors in settings where rapid disease control is needed. However, differences in trial design, including background therapy use, should be considered when interpreting these findings.

Secondary efficacy outcomes, including IGA 0/1 and EASI-90, were consistent with EASI 75 results, indicating consistency across clinician-reported measures. Patient-reported outcomes, such as SCORAD and DLQI, also favored active treatment, with meaningful improvements seen across therapies, although variability in reporting limited formal analysis. DLQI results varied between trials, likely due to differences in study design, background therapy, and the subjective nature of patient-reported measures. The relatively high placebo response rates further suggest that DLQI may be influenced by non-specific treatment effects and patient expectations. These findings emphasize the importance of interpreting patient-reported outcomes alongside objective clinical endpoints, such as EASI and IGA, and support the need for more standardized outcome reporting.

Safety profiles were acceptable during the short-term trial periods. Serious adverse events were rare, and discontinuations due to adverse events varied more widely across therapies. Importantly, no treatment-related deaths or events corresponding to the boxed-warning categories (e.g., major cardiovascular events, malignancy, VTE) were observed during trial periods. Clear patterns emerged within each class. IL-4/IL-13-pathway biologics (e.g., dupilumab, tralokinumab) were more frequently associated with conjunctivitis and nasopharyngitis, whereas JAK inhibitors showed a broader systemic adverse event profile, including acne, nausea, and laboratory abnormalities, especially at higher doses. Occasional herpes simplex/zoster events occurred with JAK inhibitors, mostly mild to moderate in severity, and rarely led to discontinuation. These patterns are consistent with their mechanisms of action: targeted cytokine blockade in biologics versus broader intracellular signaling inhibition with JAK inhibitors (20–22).

Despite the generally favorable short-term safety profile seen in RCTs, regulatory notices from the European Medicines Agency and the U.S. Food and Drug Administration have pointed out potential class risks linked to JAK inhibitors. These include higher risks of major adverse cardiovascular events, cancer, venous thromboembolism, and serious infections, especially in high-risk groups (23, 24). Such warnings mainly come from long-term safety data in other inflammatory conditions and might not be fully evident in shorter dermatology trials. Slightly higher discontinuation rates observed in some studies could reflect these broader safety concerns. Therefore, careful patient selection and continuous monitoring are essential, particularly for patients with pre-existing cardiometabolic or malignancy risk factors. Although no consistent signals for venous thromboembolism or cancer emerged within the trial periods, RCTs remain underpowered to detect rare adverse events (25). Consequently, long-term extension studies and real-world data are important to better understand delayed or infrequent adverse effects.

Strengths of this review include a protocol-driven approach, duplicate screening and extraction, inclusion limited to Level 2 evidence RCTs, and generally low risk of bias as assessed by RoB 2. Limitations include the absence of a meta-analysis due to heterogeneity in dosing, study design, and outcome timing, as well as reliance on non-responder imputation in several trials and relatively short follow-up durations. Cross-drug efficacy comparisons presented throughout this review are based on study-level, non-adjusted response rates rather than statistically validated indirect comparisons and should therefore be interpreted as descriptive rather than confirmatory. All outcomes other than EASI 75 were reported inconsistently across included trials, which was the basis for classifying them as secondary, limiting their comparability relative to the primary outcome. The search strategy and English-language restriction may also have introduced selection bias. Certainty of evidence was not formally graded using GRADE and given the narrative nature of the synthesis, we prioritized transparent reporting of study-level heterogeneity and risk of bias over formal certainty ratings. Formal assessment of reporting bias was also not performed, given the absence of meta-analysis. The adult-only eligibility criterion also excluded several pivotal mixed-age trials for the newer therapies (**Supplementary Table S3**), leaving their evidence base comparatively thinner than that of older, more established agents. This effect is uneven: the four most recently approved agents, upadacitinib, abrocitinib, lebrikizumab, and nemolizumab, each lost at least one pivotal trial to this criterion, while dupilumab and tralokinumab were largely unaffected. Additional limitations include potential confounding caused by differences in background therapy use across trials and limited synthesis of patient-reported outcomes, such as itch, sleep, and quality of life.

These findings support a personalized approach to treatment selection. Biologics trials tended to report consistent effectiveness with a well-characterized tolerability profile, whereas JAK inhibitors provide quick, dose-dependent improvements. These descriptive patterns may inform, but do not establish, comparative treatment selection. Treatment choice should consider background TCS use, desired speed of response, route of administration (oral vs injectable), comorbidities and monitoring requirements, and patient preferences.

Greater standardization in trial design and outcome reporting is needed to enable more meaningful comparisons across trials. Future research should focus on real-world studies and direct comparative trials with longer follow-up, alongside the integration of both patient- and clinician-reported outcomes. Finally, biomarker-guided selection and combination strategies (e.g., dual biologic or biologic–topical regimens) may further support the development of personalized care.

## 5 Conclusion

Across 25 RCTs, EMA-approved systemic therapies demonstrated clinically meaningful improvements in disease severity among adults with moderate-to-severe atopic dermatitis. At EMA-recommended doses, both biologics and JAK inhibitors showed substantial short-term efficacy, with higher-dose JAK inhibitors achieving particularly high response rates in some trials. Safety profiles were generally acceptable across drug classes, with predictable class-specific adverse event patterns. However, given the heterogeneity across trials and limited long-term data, treatment selection should be individualized, considering efficacy, safety, speed of response, comorbidities, monitoring needs, and patient preferences. Further long-term, standardized, and head-to-head studies, along with real-world data, are needed to improve treatment sequencing and optimize clinical outcomes.

## Data Availability

The original contributions presented in the study are included in the article/**Supplementary Material**. Further inquiries can be directed to the corresponding authors.
