# Supplementary material for: Comparative efficacy and safety of systemic therapies for moderate-to-severe atopic dermatitis in adults: a systematic review of randomized controlled trials
Source: Front Immunol. 2026 Oct 30;17:1985846. doi: 10.3389/fimmu.2026.1985846 (PMC13759417; doi:10.3389/fimmu.2026.1985846)
Supplement: Supplementary file 1 [file DataSheet1.docx]

Supplementary Material

# 1 Supplementary Data

Supplementary Material 1: Search String

Searches were conducted on 23 February 2025 across PubMed/MEDLINE, Embase, and Web of Science. Searches were limited to English-language publications.

Pubmed/MEDLINE:

("atopic dermatitis"[MeSH] OR "eczema"[Title/Abstract]) AND ("moderate"[Title/Abstract] OR "severe"[Title/Abstract]) AND ("dupilumab" OR "tralokinumab" OR "lebrikizumab" OR "nemolizumab" OR "upadacitinib" OR "abrocitinib" OR "baricitinib" OR "JAK inhibitor" OR "biologic"[Title/Abstract]) AND ("efficacy" OR "safety" OR "adverse events" OR " treatment outcome"[Title/Abstract])

EMBASE:

('atopic dermatitis'/exp OR 'eczema'/exp) AND ('moderate atopic dermatitis' OR 'severe atopic dermatitis'/exp OR ('moderate' NEAR/3 'atopic dermatitis') OR ('severe' NEAR/3 'atopic dermatitis')) AND ('dupilumab'/exp OR 'tralokinumab'/exp OR 'lebrikizumab'/exp OR 'nemolizumab'/exp OR 'upadacitinib'/exp OR 'abrocitinib'/exp OR 'baricitinib'/exp OR 'jak inhibitor'/exp OR 'biologic therapy'/exp) AND ('efficacy'/exp OR 'safety'/exp OR 'treatment outcome'/exp OR 'adverse events'/exp) AND [humans]/lim AND [adult]/lim AND [english]/lim AND [article]/lim

Web Of Science:

TS=("atopic dermatitis" OR eczema)

AND TS=("moderate atopic dermatitis" OR "severe atopic dermatitis" OR ("moderate" NEAR/5 "dermatitis") OR ("severe" NEAR/5 "dermatitis"))

AND TS=(dupilumab OR tralokinumab OR lebrikizumab OR nemolizumab OR upadacitinib OR abrocitinib OR baricitinib OR "JAK inhibitor" OR biologic)

AND TS=(efficacy OR safety OR "treatment outcome" OR "adverse events")

Supplementary Material 2: Inclusion and Exclusion Criteria

Inclusion and Exclusion Criteria:

Inclusion Criteria:

Studies qualified for inclusion according to the following criteria:

1. Study Design: Only full-text, peer-reviewed journal articles of randomized controlled trials (RCTs) were included.
2. Population:
   Studies had to include adult patients aged ≥18 years with a clinical diagnosis of moderate-to-severe atopic dermatitis (AD). Studies that included both adolescents and adults were considered only if separate data for the adult population were available.
3. Intervention:
   Eligible interventions included systemic therapies that are currently approved by the European Medicines Agency (EMA) for moderate-to-severe AD. These included:

- Biologics: Dupilumab, Tralokinumab, Lebrikizumab, Nemolizumab
- Janus Kinase (JAK) Inhibitors: Upadacitinib, Abrocitinib, Baricitinib

Both monotherapy and combination treatments involving topical anti-inflammatory agents (e.g., corticosteroids) were eligible.

4. Comparator:
   Studies were included if they compared interventions to placebo, active comparators (e.g., biologic vs. JAK inhibitor), or standard care (e.g., systemic corticosteroids).
5. Outcomes Reported:
   Included studies had to report at least one validated efficacy outcome, such as:

- Eczema Area and Severity Index (EASI)
- Investigator’s Global Assessment (IGA)
- SCORing Atopic Dermatitis (SCORAD)

Additionally, studies were required to report at least one safety outcome, such as:

- Adverse events (total, serious, or treatment-related)
- Infections (e.g., URTI, herpes simplex/zoster, conjunctivitis)
- Serious outcomes including venous thromboembolism (VTE), cardiovascular events, hepatic or hematologic abnormalities, autoimmune conditions, or malignancies

Secondary outcomes including treatment discontinuation, adherence rates, or durability of response were also considered.

6. Language and Accessibility:
   Only full-text, peer-reviewed journal studies available in English were included.

Exclusion criteria:

Studies were excluded for the following reasons:

1. Study Design:

- Non-randomized studies (e.g., retrospective cohort studies, observational studies, registry-based analyses)
- Case reports, case series, narrative reviews, systematic reviews, and meta-analyses
- Conference abstracts, editorials, unpublished data, and clinical trial protocols without published results.

2. Population:

- Studies including only pediatric patients (<18 years), unless adult data were available separately.
- Studies mainly concentrated on mild AD without a clearly identified moderate-to-severe subgroup.

3. Intervention:

- Studies evaluating non-systemic treatments such as topical corticosteroids, calcineurin inhibitors, emollients, or phototherapy.
- Studies of systemic agents not currently approved by the EMA or those involving investigational drugs without regulatory approval

4. Comparator:

- Studies lacking a clearly defined comparator group, unless reporting relevant single-arm efficacy and safety results.

5. Outcomes Reported:

- Studies not reporting clinical efficacy or safety outcomes specific to moderate-to-severe AD.
- Studies that focus only on pharmacokinetics, pharmacodynamics, or biomarkers without including clinical endpoints.
- Studies that report only quality-of-life outcomes (e.g., PROs, DLQI) without including clinical data

6. Language and Accessibility:

- Studies not published in English
- Grey literature (for example, government reports, dissertations, unpublished manuscripts)

# 2 Supplementary Tables

| Domain | Included Studies | Excluded Studies |
| --- | --- | --- |
| Study design | Randomized controlled trials (RCTs), full-text, peer-reviewed articles | Non-RCTs, review articles, conference abstracts, case series and case reports |
| Population | Adults (≥18 years) with moderate-severe AD | Pediatric-only studies without separate adult data |
| Intervention | EMA-approved targeted systemic therapies (biologics, JAK inhibitors) | Non-systemic therapies or unapproved investigational treatments |
| Comparator | Placebo, standard therapy, or active comparator | No comparator or unclear comparator |
| Outcomes | Clinical efficacy (EASI, IGA, SCORAD, DLQI) and safety (AEs, SAEs, discontinuations) | Studies without clinical efficacy or safety outcomes reported |

**Supplementary Table S1.** Summary of eligibility criteria for the systematic review. AD, atopic dermatitis. AE, adverse event. SAE, serious adverse event. EASI, Eczema Area and Severity Index. IGA, Investigator's Global Assessment. SCORAD, SCORing Atopic Dermatitis. DLQI, Dermatology Life Quality Index. EMA, European Medicines Agency. JAK, Janus kinase. RCT, randomized controlled trial. Studies enrolling combined adolescent and adult populations without adult-specific data were also excluded under the population criterion; see Supplementary Table S3 for a list of potentially eligible mixed-age trials excluded on this basis.

**Supplementary Table S2.** The summary of the study characteristics for all the included randomized controlled trials (RCTs) assessing advanced therapies for moderate-to-severe atopic dermatitis. Data include the trial phase, sample size (N), study setting/country, interventions, comparators, treatment duration in weeks, monotherapy or combination use, primary outcome measures, level of evidence (Oxford Centre for Evidence-Based Medicine, 2011), and overall risk of bias based on the Cochrane RoB 2 tool. Approval dates were obtained from the European public assessment reports (EPARs) (1).

| **Study ID** | **First Author (Year)** | **Trial Phase** | **Sample Size (N)** | **Setting/Country** | **Intervention** | **EMA approval dates** | **Comparator** | **Duration (wks)** | **Mono/Combo** | **Primary Outcome** | **Level of Evidence** | **Risk of Bias** |
| --- | --- | --- | --- | --- | --- | --- | --- | --- | --- | --- | --- | --- |
| JADE COMPARE | Bieber (2021) | Phase 3 | 838 | Global (18 countries) | Abrocitinib, Dupilumab | December 2021 (Abro), 2017 (Dupi) | Placebo and active (Dupilumab) | 16 | Combination | IGA, EASI-75 | Level 2 | Some concerns |
| Zhao et al., 2022 | Zhao (2022) | Phase 3 | 165 | China | Dupilumab | 2017 | Placebo | 16 | Monotherapy | IGA | Level 2 | Some concerns |
| TREBLE | Simpson (2018) | Phase 2 | 209 | Global (62 centers) | Lebrikizumab | November 2023 | Placebo | 12 | Combination | EASI-50 | Level 2 | Some concerns |
| ECZTRA 3 | Silverberg (2021) | Phase 3 | 380 | Europe, North America (63 centers) | Tralokinumab | June 2021 | Placebo | 16 | Combination | EASI 75, IGA 0/1 | Level 2 | Some concerns |
| Gooderham et al., 2019 | Gooderham (2019) | Phase 2b | 267 | Australia, Canada, Germany, Hungary, USA (58 centers) | Abrocitinib | December 2021 | Placebo | 12 | Monotherapy | IGA | Level 2 | Some concerns |
| Guttman-Yassky et al., 2020 | Guttman-Yassky (2020) | Phase 2b | 167 | Global (8 countries) | Upadacitinib | August 2021 | Placebo | 16 | Monotherapy | EASI, IGA | Level 2 | Some concerns |
| BREEZE-AD7 | Reich (2020) | Phase 3 | 329 | Multinational (Asia, Australia, Europe, South America; 68 sites) | Baricitinib | 2020 | Placebo | 16 | Combination | IGA 0/1 | Level 2 | Low risk |
| Thaçi et al., 2016 | Thaçi (2016) | Phase 2b | 380 | Global (10 countries; 91 centers) | Dupilumab | 2017 | Placebo | 16 | Monotherapy | EASI | Level 2 | Some concerns |
| Guttman-Yassky et al., 2020 | Guttman-Yassky (2020) | Phase 2b | 280 | USA (57 sites) | Lebrikizumab | November 2023 | Placebo | 16 | Monotherapy | EASI | Level 2 | Some concerns |
| Ruzicka et al., 2017 | Ruzicka (2017) | Phase 2 | 264 | USA, Europe, Japan | Nemolizumab | February 2025 | Placebo | 12 | Monotherapy | Pruritus VAS | Level 2 | Some concerns |
| LIBERTY AD CHRONOS | Blauvelt (2017) | Phase 3 | 740 | Global (14 countries; 161 centers) | Dupilumab | 2017 | Placebo | 52 | Combination | EASI | Level 2 | Some concerns |
| BREEZE-AD1 | Simpson (2020) | Phase 3 | 624 | Europe, Japan, India, Mexico, Taiwan (173 centers) | Baricitinib | 2020 | Placebo | 16 | Monotherapy | IGA | Level 2 | Some concerns |
| BREEZE-AD2 | Simpson (2020) | Phase 3 | 615 | Europe, Asia, Latin America, Australia (173 centers) | Baricitinib | 2020 | Placebo | 16 | Monotherapy | IGA | Level 2 | Some concerns |
| Heads Up trial | Blauvelt (2021) | Phase 3b | 673 | Global (22 countries; 129 centers) | Upadacitinib, Dupilumab | August 2021 (Upa), 2017 (Dupi) | Active (Dupilumab) | 24 | Monotherapy | EASI-75 + WP-NRS4 | Level 2 | Low risk |
| JADE DARE trial | Reich (2022) | Phase 3 | 727 | Global (15 countries; 151 sites) | Abrocitinib, Dupilumab | December 2021(Abro), 2017 (Dupi) | Active (Dupilumab) | 26 | Combination | EASI | Level 2 | Low risk |
| Silverberg 2020 JACI | Silverberg (2020) | Phase 2b | 226 | Global (6 countries) | Nemolizumab | February 2025 | Placebo | 24 | Combination | EASI | Level 2 | Some concerns |
| DUPISTAD | Merola (2023) | Phase 4 | 188 | Global (10 countries; 42 centers) | Dupilumab | 2017 | Placebo | 12 | Combination | EASI, SCORAD | Level 2 | Low risk |
| Guttman-Yassky et al., 2019 | Guttman-Yassky (2019) | Phase 2 | 124 | USA and Japan (13 centers) | Baricitinib | 2020 | Placebo | 16 | Combination | EASI-50 | Level 2 | Some concerns |
| SOLO 1 | Simpson (2016) | Phase 3 | 671 | North America, Europe, Asia | Dupilumab | 2017 | Placebo | 16 | Monotherapy | IGA | Level 2 | Some concerns |
| SOLO 2 | Simpson (2016) | Phase 3 | 708 | North America, Europe, Asia | Dupilumab | 2017 | Placebo | 16 | Monotherapy | IGA | Level 2 | Some concerns |
| ECZTRA 1 | Wollenberg (2021) | Phase 3 | 802 | France, Germany, Japan, Spain, USA | Tralokinumab | June 2021 | Placebo | 16 | Monotherapy | EASI, IGA | Level 2 | Low risk |
| ECZTRA 2 | Wollenberg (2021) | Phase 3 | 794 | France, Germany, Japan, Spain, USA | Tralokinumab | June 2021 | Placebo | 16 | Monotherapy | EASI, IGA | Level 2 | Some concerns |
| ECZTRA 7 | Gutermuth (2022) | Phase 3 | 277 | Europe (68 centers) | Tralokinumab | June 2021 | Placebo | 26 | Combination | EASI | Level 2 | Some concerns |
| LIBERTY AD CAFÉ | de Bruin-Weller (2018) | Phase 3 | 325 | Europe (10 countries) | Dupilumab | 2017 | Placebo | 16 | Combination | EASI-75 | Level 2 | Some concerns |
| BREEZE-AD4 | Bieber (2022) | Phase 3 | 463 | Global (14 countries; 102 sites) | Baricitinib | 2020 | Placebo | 52 | Combination | EASI-75 | Level 2 | Some concerns |

**Supplementary Table S3.** Pivotal mixed-age phase 3 trials excluded from this review under the adult-only population criterion. These trials were identified as pivotal phase 3 evidence for the corresponding therapies but did not meet this review’s adult-only population criterion, as they enrolled combined adolescent (typically ≥12 years) and adult populations without adult-specific results extracted for this review. Their exclusion results in a comparatively thinner evidence base for the more recently approved therapies (upadacitinib, abrocitinib, lebrikizumab, nemolizumab) relative to older, more established agents. NCT, ClinicalTrials.gov identifier.

**Supplementary Table S4.** Baseline demographic and disease characteristics of participants of the included randomized controlled trials. Data in this table include the mean age (with standard deviation), percentage of female participants, baseline disease severity scores (EASI, IGA, SCORAD), the severity criteria used for trial eligibility and reported comorbidities. NR, not reported. BSA, body surface area. TCS, topical corticosteroids. CA, ciclosporin A. HADS, Hospital Anxiety and Depression Scale.

| **Study ID / Author (Year)** | **Mean Age (± SD)** | **% Female** | **Baseline Severity Score (EASI / IGA / SCORAD)** | **Baseline Severity Criteria Used** | **Comorbidities Reported** |
| --- | --- | --- | --- | --- | --- |
| JADE COMPARE | 37.7 ± 14.7 | 51.1% | EASI: 32.5 ± 11.6; IGA 3: 64.6%, IGA 4: 35.3%; SCORAD: 67.9 ± 12.6 | EASI ≥16, IGA ≥3, ≥10% BSA, PP-NRS ≥4 | Asthma: 33.9%, Allergic conjunctivitis: 9.4%, Food allergy: 14.9% |
| Zhao et al., 2022 | 28.0 ± 12.6* | 28.5% | EASI: 30.65 ± 13.10*,  IGA 4: 56.36%,  SCORAD: NR | EASI ≥16, IGA ≥3, BSA ≥10% | Allergic rhinitis (53.9%), asthma (21.8%), allergic conjunctivitis (10.3%) |
| TREBLE | 36.1 ± 9.63* | 35.0% | EASI: 25.34 ± 11.10*  IGA: 3.23 ± 0.43  SCORAD: 58.84 ± 13.13* | EASI ≥14, IGA ≥3, BSA ≥10%, Pruritus VAS ≥3 | Not specified. Other skin conditions excluded; included TCS-refractory disease and eosinophilic involvement |
| ECZTRA 3 | 36.0 ± 18.27* | 45.0% | EASI: 25.5 ± 13.26*  IGA 3: 53.2%, IGA 4: 46.3%  SCORAD: 66.5 ± 14.59* | Moderate: IGA 3, Severe: IGA 4 | Allergic conjunctivitis 22.1%, asthma 46.6%, food allergy 36.3%, hay fever 55.3%. Atopic keratoconjunctivitis and eczema herpeticum reported infrequently |
| Gooderham et al., 2019 | 40.8 ± 16.1 | 46.1% | EASI: 25.37 ± 12.12  IGA 3: 52.7–63.0%, IGA 4: 37.0–47.3%;  SCORAD: 64.17 ± 13.12 | EASI ≥16, IGA ≥3, ≥10% BSA, chronic AD ≥3 yrs, inadequate response/contraindication to topicals | Asthma: 3.0–6.0%; other allergic diseases not specified. Skin disease exclusions applied |
| Guttman-Yassky et al., 2020  (Upadacitinib) | 39.95 ± 15.76 | 37.7% | EASI: 30.89 ± 13.62  IGA 3: 58.08%, IGA 4: 41.92%  SCORAD: NR | EASI ≥16, IGA ≥3, ≥10% BSA, chronic AD ≥3 yrs, inadequate response to topical therapy | History of asthma: 38% across all groups |
| BREEZE-AD7 | 33.8 ± 12.4 | 34.0% | EASI: 29.57 ± 12.28  IGA 4: 46.39%  SCORAD: 67.24 ± 13.65 | EASI ≥16, vIGA-AD ≥3, ≥10% BSA, inadequate response/intolerance to TCS | Depression (~8.2%), sleep disturbance (avg. 2.5 awakenings/night); other comorbidities reported via PROMs, not numerically specified |
| **Thaçi et al., 2016** | 37.0 ± 12.1 | 48.8% | EASI: 31.87 ± 13.41  IGA 3: 52.77%, IGA 4: 47.23%  SCORAD: 67.39 ± 13.28 | EASI ≥16, IGA ≥3, ≥10% BSA, chronic AD ≥3 yrs, inadequate response to topical therapy | Headache (11%), herpes infections (8%), URTIs (37%), skin/soft tissue infections (7%), UTIs (6%), AD flares (20%) |
| Guttman-Yassky et al., 2020  (lebrikizumab) | 39.3 ± 17.5 | 59.3% | EASI: 27.48 ± 11.75  IGA 3: 65.00%, IGA 4: 35.00%  SCORAD: NR | EASI ≥16, IGA ≥3, ≥10% BSA, chronic AD ≥1 yr, inadequate response/intolerance to topical therapies | No detailed comorbidities listed in primary publication |
| Ruzicka et al., 2017 | 35.3 ± 10.7 | 56.8% | EASI: 29.44 ± 14.49  IGA 3: 46.2%, IGA 4: 42.4%, IGA 5: 11.4%;  SCORAD: NR | EASI ≥10, Pruritus VAS ≥50mm, sIGA ≥3, inadequate response to TCS/calcineurin inhibitors; moderate-to-severe, chronic and active at baseline | Allergic rhinitis (31–39%), conjunctivitis (11–25%), food allergy (9–27%), asthma (4–14%), hypertension (5–11%) |
| LIBERTY AD CHRONOS | 34.93 ± 14.60 | 39.7% | EASI: 29.53 ± 14.01  IGA 3: 53.0%, IGA 4: 47.0%; SCORAD: 65.42 ± 15.17 | IGA ≥3, EASI ≥16, disease duration ≥3 yrs, prior inadequate response to topical/systemic therapy within 6 months | Allergies (63%), rhinitis (41–48%), asthma (37–41%), food allergy (30–36%), conjunctivitis (22–28%) |
| BREEZE-AD1 | 35.60 ± 12.84 | 39.1% | EASI: 31.19 ± 12.48  IGA 4: 41.83%  SCORAD: 67.59 ± 13.67 | EASI ≥16, vIGA-AD ≥3, BSA ≥10% | Not explicitly reported by %: herpes simplex history part of exclusion criteria |
| BREEZE-AD2 | 34.59 ± 12.74 | 36.9% | EASI: 33.40 ± 13.45  IGA 4: 50.24%  SCORAD: 68.00 ± 13.03 | EASI ≥16, vIGA-AD ≥3, BSA ≥10% | Not explicitly reported: exclusions included recent eczema herpeticum, VTE/MACE, or immunodeficiency |
| Heads Up trial | 36.05 ± 14.1 | 44.30% | EASI: 30.11 ± 12.14  IGA: IGA 3: 48.74%, IGA 4: 51.26%  SCORAD: NR | EASI ≥ 16, IGA ≥ 3, ≥10% BSA affected, Weekly Worst Pruritus NRS ≥ 4 | Not specified; moderate-to-severe AD eligible for systemic therapy, comorbidities not reported |
| JADE DARE trial | 36.3 ± 13.96 | 46% | EASI: 28.10 ± 11.69  IGA: IGA 3: 59.97%, IGA 4: 40.03%  SCORAD: NR | EASI ≥16, IGA ≥3, PP-NRS ≥4, BSA ≥10% | Asthma: Abrocitinib = 28%, Dupilumab = 35%  Allergic Rhinitis: Abrocitinib = 17%, Dupilumab = 19% |
| Silverberg 2020 JACI | 39.3 ± 15.45 | 49.10% | EASI: 25.48 ± 10.92  IGA: IGA 3: 66.8%, IGA 4: 33.2%  SCORAD: 66.74 ± 11.57 | EASI ≥12, IGA ≥3, BSA ≥10%, Pruritus NRS ≥7, documented inadequate control with topical treatment in past 6 months | Asthma in pre-existing cases (up to 17.5%, dose-dependent); no de novo asthma, autoimmune conditions, or malignancies reported |
| DUPISTAD | 35.7 ± 14.9 | 51.60% | EASI: 26.14 ± 11.26  IGA: IGA 3: 65.4%, IGA 4: 34.6%  SCORAD: 64.08 ± 12.50 | EASI score ≥12, PP NRS score ≥3, Sleep NRS score ≥5, inadequate response to topical AD treatments | Not reported / major comorbidities and psychiatric conditions excluded |
| Guttman-Yassky et al., 2019 | 37.2 ±16.7* | 45.2% | EASI 21.6 ± 10.0*  IGA: 3 (IQR 3–4)  SCORAD: 55.4 ± 11.7* | EASI ≥12, BSA >10%, disease ≥2 years, inadequate response to emollients and topical/systemic therapies | Not fully reported; key comorbidities (e.g. uncontrolled asthma, autoimmune, psychiatric) excluded — likely low-comorbidity population |
| SOLO 1 | 38.67 ± 16.79 | 42% | EASI: 30.67 ± 14.84  IGA: IGA 4: 48.29%  SCORAD: 66.00 ± 14.59 | IGA 3–4, AD ≥3 yrs (AAD criteria), inadequate response or contraindication to topical therapy | HADS ≥8 in 43% (97 patients in placebo arm) |
| SOLO 2 | 34.67 ± 15.80 | 41% | EASI: 29.37 ± 14.65  IGA: IGA 4: 48.31%  SCORAD: 68.03 ± 14.52 | IGA 3–4, AD ≥3 yrs (AAD criteria), inadequate response or contraindication to topical therapy | HADS ≥8 in 40–43% (e.g. 95 placebo patients = 40%) |
| ECZTRA 1 | 37.00 ± 15.92 | 40.90% | EASI: 28.72 ± 14.00  IGA: IGA 4: 50.8%  SCORAD: 69.60 ± 12.96 | IGA ≥3, EASI ≥16, BSA ≥10%, pruritus NRS ≥4 | Not detailed; prior treatment common — ~60–70% systemic steroids, ~30–38% ciclosporin |
| ECZTRA 2 | 32.99 ± 17.04 | 40.40% | EASI: 28.55 ± 15.52  IGA: IGA 4: 48.7%  SCORAD: 69.60 ± 13.52 | IGA ≥3, EASI ≥16, BSA ≥10%, pruritus NRS ≥4 | Not detailed; prior treatment included TCS (98–100%), systemic steroids (59–69%), ciclosporin (32–38%) |
| ECZTRA 7 | 34.0 (IQR 26.0–45.0), median | 40.4% | EASI: 28.8 (IQR 22.4–39.6), median  IGA: NR  SCORAD: 69.1 (IQR 61.4–78.9), median | EASI ≥ 20, IGA ≥ 3, Pruritus NRS ≥ 4, BSA involvement ≥ 10% | Not quantified, long-standing AD common. Prior treatment: ~70% systemic steroids, 17–19% methotrexate, ~13% azathioprine, ~5% dupilumab |
| LIBERTY AD CAFÉ | 37.83 ± 15.05 | 38.8% | EASI: 31.46 ± 11.11  IGA: IGA 4: 47.4%  SCORAD: 66.76 ± 13.15 | EASI ≥ 20, IGA ≥ 3, ≥10% Body Surface Area affected | High allergic comorbidity burden: rhinitis (~56%), asthma (~38–46%), conjunctivitis (~36–55%), food allergy (~38–49%), others (~66–71%) |
| BREEZE-AD4 | 38.2 ± 13.6 | 36% | EASI: 31.82 ± 12.83  IGA: IGA 4: 51%  SCORAD: 68.76 ± 13.44 | EASI ≥16, vIGA-AD ≥3, BSA ≥10%, history of inadequate response to TCS, and inadequate response, contraindication or intolerance to CA | Not detailed; ~62–70% had prior ciclosporin use, indicating refractory/intolerant disease |

*: Mean (SD) estimated from arm-level medians (IQR) using Wan et al. (2). Original source reported per-arm medians only.

**Supplementary Table S5.** EASI 75 response rates (%) reported at all tested doses across all the included randomized controlled trials. The table shows the proportion of patients in each treatment group who achieved at least a 75% improvement from baseline in EASI score. QD, once daily. QW, once weekly. Q2W, every two weeks. Q4W, every 4 weeks. TCS, topical corticosteroids.

| **Study ID** | **Drug** | **Dose** | **Week** | **EASI-75 (%)** |
| --- | --- | --- | --- | --- |
| JADE COMPARE | Abrocitinib | 200 mg QD | 16 | 71.0 |
| JADE COMPARE | Abrocitinib | 100 mg QD | 16 | 60.3 |
| JADE COMPARE | Dupilumab | 300 mg Q2W | 16 | 65.5 |
| JADE COMPARE | Placebo | — | 16 | 30.6 |
| Zhao et al., 2022 | Dupilumab | 300 mg Q2W | 16 | 57.3 |
| Zhao et al., 2022 | Placebo | — | 16 | 14.5 |
| TREBLE | Lebrikizumab | 125 mg Q4W | 12 | 54.9 |
| TREBLE | Lebrikizumab | 125 mg single dose | 12 | 38.5 |
| TREBLE | Lebrikizumab | 250 mg single dose | 12 | 49.1 |
| TREBLE | Placebo | — | 12 | 34.0 |
| ECZTRA 3 | Tralokinumab | 300 mg Q2W | 16 | 56.0 |
| ECZTRA 3 | Placebo | — | 16 | 35.7 |
| Gooderham et al., 2019 | Abrocitinib | 10 mg QD | 12 | 17.4 |
| Gooderham et al., 2019 | Abrocitinib | 30 mg QD | 12 | 13.3 |
| Gooderham et al., 2019 | Abrocitinib | 100 mg QD | 12 | 40.7 |
| Gooderham et al., 2019 | Abrocitinib | 200 mg QD | 12 | 64.6 |
| Gooderham et al., 2019 | Placebo | — | 12 | 15.4 |
| Guttman-Yassky et al., 2020 | Upadacitinib | 7.5 mg QD | 16 | 24 |
| Guttman-Yassky et al., 2020 | Upadacitinib | 15 mg QD | 16 | 59 |
| Guttman-Yassky et al., 2020 | Upadacitinib | 30 mg QD | 16 | 73 |
| Guttman-Yassky et al., 2020 | Placebo | — | 16 | 12 |
| BREEZE-AD7 | Baricitinib | 2 mg QD | 16 | 43 |
| BREEZE-AD7 | Baricitinib | 4 mg QD | 16 | 48 |
| BREEZE-AD7 | Placebo | — | 16 | 23 |
| Thaçi et al., 2016 | Dupilumab | 300 mg QW | 16 | 60.3 |
| Thaçi et al., 2016 | Placebo | — | 16 | 11.5 |
| Guttman-Yassky et al., 2020 | Lebrikizumab | 125 mg Q4W | 16 | 43.3 |
| Guttman-Yassky et al., 2020 | Lebrikizumab | 250 mg Q4W | 16 | 56.1 |
| Guttman-Yassky et al., 2020 | Lebrikizumab | 250 mg Q2W | 16 | 60.6 |
| Guttman-Yassky et al., 2020 | Placebo | — | 16 | 24.3 |
| Ruzicka et al., 2017 | Nemolizumab | 0.1 mg/kg Q4W | 12 | 32.3 |
| Ruzicka et al., 2017 | Nemolizumab | 0.5 mg/kg Q4W | 12 | 37.7 |
| Ruzicka et al., 2017 | Nemolizumab | 2.0 mg/kg Q4W | 12 | 22.3 |
| Ruzicka et al., 2017 | Placebo | — | 12 | 18.3 |
| LIBERTY AD CHRONOS | Dupilumab | 300 mg QW | 16 | 64 |
| LIBERTY AD CHRONOS | Dupilumab | 300 mg Q2W | 16 | 69 |
| LIBERTY AD CHRONOS | Placebo | — | 16 | 23 |
| BREEZE-AD1 | Baricitinib | 1 mg QD | 16 | 17.3 |
| BREEZE-AD1 | Baricitinib | 2 mg QD | 16 | 18.7 |
| BREEZE-AD1 | Baricitinib | 4 mg QD | 16 | 24.8 |
| BREEZE-AD1 | Placebo | — | 16 | 8.8 |
| BREEZE-AD2 | Baricitinib | 1 mg QD | 16 | 12.8 |
| BREEZE-AD2 | Baricitinib | 2 mg QD | 16 | 17.9 |
| BREEZE-AD2 | Baricitinib | 4 mg QD | 16 | 21.1 |
| BREEZE-AD2 | Placebo | — | 16 | 6.1 |
| Heads Up trial | Upadacitinib | 30 mg QD | 16 | 72.4 |
| Heads Up trial | Dupilumab | 300 mg Q2W | 16 | 62.6 |
| JADE DARE trial | Abrocitinib | 200 mg QD | 16 | 77.3 |
| JADE DARE trial | Dupilumab | 300 mg Q2W | 16 | 67.8 |
| Silverberg 2020 JACI | Nemolizumab | 30 mg Q4W | 16 | 49.1 |
| Silverberg 2020 JACI | Placebo | — | 16 | 19.3 |
| DUPISTAD | Dupilumab | 300 mg Q2W | 12 | 60.6 |
| DUPISTAD | Placebo | — | 12 | 29.2 |
| Guttman-Yassky et al., 2019 | Baricitinib | 2 mg QD | 16 | 30 |
| Guttman-Yassky et al., 2019 | Baricitinib | 4 mg QD | 16 | 34 |
| Guttman-Yassky et al., 2019 | Placebo | — | 16 | 20 |
| SOLO 1 | Dupilumab | 300 mg Q2W | 16 | 51 |
| SOLO 1 | Dupilumab | 300 mg QW | 16 | 52 |
| SOLO 1 | Placebo | — | 16 | 15 |
| SOLO 2 | Dupilumab | 300 mg Q2W | 16 | 44 |
| SOLO 2 | Dupilumab | 300 mg QW | 16 | 48 |
| SOLO 2 | Placebo | — | 16 | 12 |
| ECZTRA 1 | Tralokinumab | 300 mg Q2W | 16 | 25.0 |
| ECZTRA 1 | Placebo | — | 16 | 12.7 |
| ECZTRA 2 | Tralokinumab | 300 mg Q2W | 16 | 33.2 |
| ECZTRA 2 | Placebo | — | 16 | 11.4 |
| ECZTRA 7 | Tralokinumab | 300 mg Q2W | 16 | 64.2 |
| ECZTRA 7 | Placebo | — | 16 | 50.5 |
| LIBERTY AD CAFÉ | Dupilumab | 300 mg Q2W + TCS | 16 | 62.6 |
| LIBERTY AD CAFÉ | Dupilumab | 300 mg QW + TCS | 16 | 59.1 |
| LIBERTY AD CAFÉ | Placebo | — | 16 | 29.6 |
| BREEZE-AD4 | Baricitinib | 1 mg QD + TCS | 16 | 33 |
| BREEZE-AD4 | Baricitinib | 2 mg QD + TCS | 16 | 30 |
| BREEZE-AD4 | Baricitinib | 4 mg QD + TCS | 16 | 37 |
| BREEZE-AD4 | Placebo + TCS | — | 16 | 27 |

**Supplementary Table S6.** EASI-90 response rates (%) reported at all tested doses across all included randomized controlled trials. The table shows the percentage of patients in each treatment group who achieve at least a 90% improvement from baseline in EASI score, representing a higher-threshold efficacy outcome in moderate-to-severe atopic dermatitis. QD, once daily, QW, once weekly. Q2W, every 2 weeks. Q4W, every 4 weeks. TCS, topical corticosteroids. NR, not reported.

| **Study ID** | **Drug** | **Dose** | **Week** | **EASI-90 (%)** |
| --- | --- | --- | --- | --- |
| JADE COMPARE | Abrocitinib | 200 mg QD | 16 | 48.9 |
| JADE COMPARE | Abrocitinib | 100 mg QD | 16 | 38.0 |
| JADE COMPARE | Dupilumab | 300 mg Q2W | 16 | 38.8 |
| JADE COMPARE | Placebo | — | 16 | 11.3 |
| Zhao et al., 2022 | Dupilumab | 300 mg Q2W | 16 | 40.2 |
| Zhao et al., 2022 | Placebo | — | 16 | 6.0 |
| TREBLE | Lebrikizumab | 125 mg Q4W | 12 | NR |
| TREBLE | Lebrikizumab | 125 mg single dose | 12 | NR |
| TREBLE | Lebrikizumab | 250 mg single dose | 12 | NR |
| TREBLE | Placebo | — | 12 | NR |
| ECZTRA 3 | Tralokinumab | 300 mg Q2W | 16 | 32.9 |
| ECZTRA 3 | Placebo | — | 16 | 21.4 |
| Gooderham et al., 2019 | Abrocitinib | 10 mg QD | 12 | 10.9 |
| Gooderham et al., 2019 | Abrocitinib | 30 mg QD | 12 | 0.0 |
| Gooderham et al., 2019 | Abrocitinib | 100 mg QD | 12 | 25.9 |
| Gooderham et al., 2019 | Abrocitinib | 200 mg QD | 12 | 52.1 |
| Gooderham et al., 2019 | Placebo | — | 12 | 9.6 |
| Guttman-Yassky et al., 2020 | Upadacitinib | 7.5 mg QD | 16 | 14.0 |
| Guttman-Yassky et al., 2020 | Upadacitinib | 15 mg QD | 16 | 43.0 |
| Guttman-Yassky et al., 2020 | Upadacitinib | 30 mg QD | 16 | 50.0 |
| Guttman-Yassky et al., 2020 | Placebo | — | 16 | 0.0 |
| BREEZE-AD7 | Baricitinib | 2 mg QD | 16 | 17.0 |
| BREEZE-AD7 | Baricitinib | 4 mg QD | 16 | 24.0 |
| BREEZE-AD7 | Placebo | — | 16 | 14.0 |
| Thaçi et al., 2016 | Dupilumab | 300 mg QW | 16 | 36.5 |
| Thaçi et al., 2016 | Placebo | — | 16 | 3.3 |
| Guttman-Yassky et al., 2020 | Lebrikizumab | 125 mg Q4W | 16 | 26.1 |
| Guttman-Yassky et al., 2020 | Lebrikizumab | 250 mg Q4W | 16 | 36.1 |
| Guttman-Yassky et al., 2020 | Lebrikizumab | 250 mg Q2W | 16 | 44.0 |
| Guttman-Yassky et al., 2020 | Placebo | — | 16 | 11.4 |
| Ruzicka et al., 2017 | Nemolizumab | 0.1 mg/kg Q4W | 12 | NR |
| Ruzicka et al., 2017 | Nemolizumab | 0.5 mg/kg Q4W | 12 | NR |
| Ruzicka et al., 2017 | Nemolizumab | 2.0 mg/kg Q4W | 12 | NR |
| Ruzicka et al., 2017 | Placebo | — | 12 | NR |
| LIBERTY AD CHRONOS | Dupilumab | 300 mg QW | 16 | 43.0 |
| LIBERTY AD CHRONOS | Dupilumab | 300 mg Q2W | 16 | 40.0 |
| LIBERTY AD CHRONOS | Placebo | — | 16 | 11.0 |
| BREEZE-AD1 | Baricitinib | 1 mg QD | 16 | 8.7 |
| BREEZE-AD1 | Baricitinib | 2 mg QD | 16 | 10.6 |
| BREEZE-AD1 | Baricitinib | 4 mg QD | 16 | 16.0 |
| BREEZE-AD1 | Placebo | — | 16 | 4.8 |
| BREEZE-AD2 | Baricitinib | 1 mg QD | 16 | 6.4 |
| BREEZE-AD2 | Baricitinib | 2 mg QD | 16 | 8.9 |
| BREEZE-AD2 | Baricitinib | 4 mg QD | 16 | 13.0 |
| BREEZE-AD2 | Placebo | — | 16 | 2.5 |
| Heads Up trial | Upadacitinib | 30 mg QD | 16 | 61.6 |
| Heads Up trial | Dupilumab | 300 mg Q2W | 16 | 40.3 |
| JADE DARE trial | Abrocitinib | 200 mg QD | 16 | 54.0 |
| JADE DARE trial | Dupilumab | 300 mg Q2W | 16 | 42.0 |
| Silverberg 2020 JACI | Nemolizumab | 30 mg Q4W | 16 | 33.3 |
| Silverberg 2020 JACI | Placebo | — | 16 | 8.8 |
| DUPISTAD | Dupilumab | 300 mg Q2W | 12 | 40.4 |
| DUPISTAD | Placebo | — | 12 | 14.6 |
| Guttman-Yassky et al., 2019 | Baricitinib | 2 mg QD | 16 | 19.0 |
| Guttman-Yassky et al., 2019 | Baricitinib | 4 mg QD | 16 | 25.0 |
| Guttman-Yassky et al., 2019 | Placebo | — | 16 | 6.0 |
| SOLO 1 | Dupilumab | 300 mg Q2W | 16 | 36.0 |
| SOLO 1 | Dupilumab | 300 mg QW | 16 | 33.0 |
| SOLO 1 | Placebo | — | 16 | 8.0 |
| SOLO 2 | Dupilumab | 300 mg Q2W | 16 | 30.0 |
| SOLO 2 | Dupilumab | 300 mg QW | 16 | 31.0 |
| SOLO 2 | Placebo | — | 16 | 7.0 |
| ECZTRA 1 | Tralokinumab | 300 mg Q2W | 16 | 14.5 |
| ECZTRA 1 | Placebo | — | 16 | 4.1 |
| ECZTRA 2 | Tralokinumab | 300 mg Q2W | 16 | 18.3 |
| ECZTRA 2 | Placebo | — | 16 | 5.5 |
| ECZTRA 7 | Tralokinumab | 300 mg Q2W | 16 | 41.1 |
| ECZTRA 7 | Placebo | — | 16 | 29.3 |
| LIBERTY AD CAFÉ | Dupilumab | 300 mg Q2W + TCS | 16 | 45.8 |
| LIBERTY AD CAFÉ | Dupilumab | 300 mg QW + TCS | 16 | 37.3 |
| LIBERTY AD CAFÉ | Placebo | — | 16 | 12.0 |
| BREEZE-AD4 | Baricitinib | 1 mg QD + TCS | 16 | 9.0 |
| BREEZE-AD4 | Baricitinib | 2 mg QD + TCS | 16 | 10.0 |
| BREEZE-AD4 | Baricitinib | 4 mg QD + TCS | 16 | 14.0 |
| BREEZE-AD4 | Placebo + TCS | — | 16 | 7.0 |

**Supplementary Table S7.** EASI-90 response rates (%) at EMA-recommended doses, grouped by treatment, with head-to-head trials shown separately. Only treatment arms with licensed dosing schedules are included. QD, once daily, QW, once weekly. Q2W, every 2 weeks. Q4W, every 4 weeks.

| **Study ID** | **Drug** | **Dose** | **Week** | **EASI-90 (%)** |
| --- | --- | --- | --- | --- |
| ***Head-to-head trials*** | | | | |
| JADE COMPARE | Dupilumab | 300 mg Q2W | 16 | 38.80 |
| JADE COMPARE | Abrocitinib | 200 mg QD | 16 | 48.90 |
| JADE COMPARE | Abrocitinib | 100 mg QD | 16 | 38.00 |
| JADE DARE trial | Abrocitinib | 200 mg QD | 16 | 54.00 |
| JADE DARE trial | Dupilumab | 300 mg Q2W | 16 | 42.00 |
| Heads Up trial | Upadacitinib | 30 mg QD | 16 | 61.60 |
| Heads Up trial | Dupilumab | 300 mg Q2W | 16 | 40.30 |
| ***Abrocitinib*** | | | | |
| Gooderham et al., 2019 | Abrocitinib | 100 mg QD | 12 | 25.90 |
| Gooderham et al., 2019 | Abrocitinib | 200 mg QD | 12 | 52.10 |
| ***Upadacitinib*** | | | | |
| Guttman-Yassky et al., 2020 | Upadacitinib | 15 mg QD | 16 | 43.00 |
| Guttman-Yassky et al., 2020 | Upadacitinib | 30 mg QD | 16 | 50.00 |
| ***Baricitinib*** | | | | |
| BREEZE-AD1 | Baricitinib | 2 mg QD | 16 | 10.60 |
| BREEZE-AD1 | Baricitinib | 4 mg QD | 16 | 16.00 |
| BREEZE-AD2 | Baricitinib | 2 mg QD | 16 | 8.90 |
| BREEZE-AD2 | Baricitinib | 4 mg QD | 16 | 13.00 |
| Guttman-Yassky et al., 2019 | Baricitinib | 2 mg QD | 16 | 19.00 |
| Guttman-Yassky et al., 2019 | Baricitinib | 4 mg QD | 16 | 25.00 |
| BREEZE-AD4 | Baricitinib | 2 mg QD | 16 | 10.00 |
| BREEZE-AD4 | Baricitinib | 4 mg QD | 16 | 14.00 |
| BREEZE-AD7 | Baricitinib | 2 mg QD | 16 | 17.00 |
| BREEZE-AD7 | Baricitinib | 4 mg QD | 16 | 24.00 |
| ***Dupilumab*** | | | | |
| Zhao et al., 2022 | Dupilumab | 300 mg Q2W | 16 | 40.20 |
| LIBERTY AD CHRONOS | Dupilumab | 300 mg Q2W | 16 | 40.00 |
| DUPISTAD | Dupilumab | 300 mg Q2W | 12 | 40.40 |
| SOLO 1 | Dupilumab | 300 mg Q2W | 16 | 36.00 |
| SOLO 2 | Dupilumab | 300 mg Q2W | 16 | 30.00 |
| LIBERTY AD CAFÉ | Dupilumab | 300 mg Q2W | 16 | 45.80 |
| ***Tralokinumab*** | | | | |
| ECZTRA 3 | Tralokinumab | 300 mg Q2W | 16 | 32.90 |
| ECZTRA 1 | Tralokinumab | 300 mg Q2W | 16 | 14.50 |
| ECZTRA 2 | Tralokinumab | 300 mg Q2W | 16 | 18.30 |
| ECZTRA 7 | Tralokinumab | 300 mg Q2W | 16 | 41.10 |
| ***Lebrikizumab*** | | | | |
| Guttman-Yassky et al., 2020 | Lebrikizumab | 250 mg Q2W | 16 | 44.00 |
| ***Nemolizumab*** | | | | |
| Silverberg 2020 JACI | Nemolizumab | 30 mg Q4W | 16 | 33.30 |

**Supplementary Table S8.** Investigator’s Global Assessment (IGA) 0/1 response rates (%) reported at all tested doses across all included randomized controlled QD, once daily, QW, once weekly. Q2W, every 2 weeks. Q4W, every 4 weeks. TCS, topical corticosteroids. NR, not reported.

| **Study ID** | **Drug** | **Dose** | **Week** | **IGA 0/1 (%)** |
| --- | --- | --- | --- | --- |
| JADE COMPARE | Abrocitinib | 200 mg QD | 16 | 47.5 |
| JADE COMPARE | Abrocitinib | 100 mg QD | 16 | 34.8 |
| JADE COMPARE | Dupilumab | 300 mg Q2W | 16 | 38.8 |
| JADE COMPARE | Placebo | — | 16 | 12.9 |
| Zhao et al., 2022 | Dupilumab | 300 mg Q2W | 16 | 26.8 |
| Zhao et al., 2022 | Placebo | — | 16 | 4.8 |
| TREBLE | Lebrikizumab | 125 mg Q4W | 12 | 33.3 |
| TREBLE | Lebrikizumab | 125 mg single dose | 12 | 21.2 |
| TREBLE | Lebrikizumab | 250 mg single dose | 12 | 28.3 |
| TREBLE | Placebo | — | 12 | 18.9 |
| ECZTRA 3 | Tralokinumab | 300 mg Q2W | 16 | 38.9 |
| ECZTRA 3 | Placebo | — | 16 | 26.2 |
| Gooderham et al., 2019 | Abrocitinib | 10 mg QD | 12 | 10.9 |
| Gooderham et al., 2019 | Abrocitinib | 30 mg QD | 12 | 8.9 |
| Gooderham et al., 2019 | Abrocitinib | 100 mg QD | 12 | 29.6 |
| Gooderham et al., 2019 | Abrocitinib | 200 mg QD | 12 | 43.8 |
| Gooderham et al., 2019 | Placebo | — | 12 | 5.8 |
| Guttman-Yassky et al., 2020 | Upadacitinib | 7.5 mg QD | 16 | 24.0 |
| Guttman-Yassky et al., 2020 | Upadacitinib | 15 mg QD | 16 | 43.0 |
| Guttman-Yassky et al., 2020 | Upadacitinib | 30 mg QD | 16 | 50.0 |
| Guttman-Yassky et al., 2020 | Placebo | — | 16 | 7.0 |
| BREEZE-AD7 | Baricitinib | 2 mg QD | 16 | 24.0 |
| BREEZE-AD7 | Baricitinib | 4 mg QD | 16 | 31.0 |
| BREEZE-AD7 | Placebo | — | 16 | 15.0 |
| Thaçi et al., 2016 | Dupilumab | 300 mg QW | 16 | 33.0 |
| Thaçi et al., 2016 | Placebo | — | 16 | 2.0 |
| Guttman-Yassky et al., 2020 | Lebrikizumab | 125 mg Q4W | 16 | 26.6 |
| Guttman-Yassky et al., 2020 | Lebrikizumab | 250 mg Q4W | 16 | 33.7 |
| Guttman-Yassky et al., 2020 | Lebrikizumab | 250 mg Q2W | 16 | 44.6 |
| Guttman-Yassky et al., 2020 | Placebo | — | 16 | 15.3 |
| Ruzicka et al., 2017 | Nemolizumab | 0.1 mg/kg Q4W | 12 | 14.0 |
| Ruzicka et al., 2017 | Nemolizumab | 0.5 mg/kg Q4W | 12 | 38.0 |
| Ruzicka et al., 2017 | Nemolizumab | 2.0 mg/kg Q4W | 12 | 25.0 |
| Ruzicka et al., 2017 | Placebo | — | 12 | 11.0 |
| LIBERTY AD CHRONOS | Dupilumab | 300 mg QW | 16 | 39.0 |
| LIBERTY AD CHRONOS | Dupilumab | 300 mg Q2W | 16 | 39.0 |
| LIBERTY AD CHRONOS | Placebo | — | 16 | 12.0 |
| BREEZE-AD1 | Baricitinib | 1 mg QD | 16 | 11.8 |
| BREEZE-AD1 | Baricitinib | 2 mg QD | 16 | 11.4 |
| BREEZE-AD1 | Baricitinib | 4 mg QD | 16 | 16.8 |
| BREEZE-AD1 | Placebo | — | 16 | 4.8 |
| BREEZE-AD2 | Baricitinib | 1 mg QD | 16 | 8.8 |
| BREEZE-AD2 | Baricitinib | 2 mg QD | 16 | 10.6 |
| BREEZE-AD2 | Baricitinib | 4 mg QD | 16 | 13.8 |
| BREEZE-AD2 | Placebo | — | 16 | 4.5 |
| Heads Up trial | Upadacitinib | 30 mg QD | 16 | NR |
| Heads Up trial | Dupilumab | 300 mg Q2W | 16 | NR |
| JADE DARE trial | Abrocitinib | 200 mg QD | 16 | 55.3 |
| JADE DARE trial | Dupilumab | 300 mg Q2W | 16 | 42.5 |
| Silverberg 2020 JACI | Nemolizumab | 30 mg Q4W | 16 | 33.3 |
| Silverberg 2020 JACI | Placebo | — | 16 | 12.3 |
| DUPISTAD | Dupilumab | 300 mg Q2W | 12 | NR |
| DUPISTAD | Placebo | — | 12 | NR |
| Guttman-Yassky et al., 2019 | Baricitinib | 2 mg QD | 16 | 22.0 |
| Guttman-Yassky et al., 2019 | Baricitinib | 4 mg QD | 16 | 21.0 |
| Guttman-Yassky et al., 2019 | Placebo | — | 16 | 8.0 |
| SOLO 1 | Dupilumab | 300 mg Q2W | 16 | 38.0 |
| SOLO 1 | Dupilumab | 300 mg QW | 16 | 37.0 |
| SOLO 1 | Placebo | — | 16 | 10.0 |
| SOLO 2 | Dupilumab | 300 mg Q2W | 16 | 36.0 |
| SOLO 2 | Dupilumab | 300 mg QW | 16 | 36.0 |
| SOLO 2 | Placebo | — | 16 | 8.0 |
| ECZTRA 1 | Tralokinumab | 300 mg Q2W | 16 | 15.8 |
| ECZTRA 1 | Placebo | — | 16 | 7.1 |
| ECZTRA 2 | Tralokinumab | 300 mg Q2W | 16 | 22.2 |
| ECZTRA 2 | Placebo | — | 16 | 10.9 |
| ECZTRA 7 | Tralokinumab | 300 mg Q2W | 16 | NR |
| ECZTRA 7 | Placebo | — | 16 | NR |
| LIBERTY AD CAFÉ | Dupilumab | 300 mg Q2W + TCS | 16 | 40.2 |
| LIBERTY AD CAFÉ | Dupilumab | 300 mg QW + TCS | 16 | 39.1 |
| LIBERTY AD CAFÉ | Placebo | — | 16 | 13.9 |
| BREEZE-AD4 | Baricitinib | 1 mg QD + TCS | 16 | 13.0 |
| BREEZE-AD4 | Baricitinib | 2 mg QD + TCS | 16 | 15.0 |
| BREEZE-AD4 | Baricitinib | 4 mg QD + TCS | 16 | 22.0 |
| BREEZE-AD4 | Placebo + TCS | — | 16 | 10.0 |

**Supplementary Table S9.** Investigator’s Global Assessment (IGA) 0/1 response rates (%) at EMA-recommended doses, grouped by treatment with head-to-head trials shown separately. Only treatment arms using licensed dosing schedules are included. QD, once daily, QW, once weekly. Q2W, every 2 weeks. Q4W, every 4 weeks. TCS, topical corticosteroids. NR, not reported.

| **Study ID** | **Drug** | **Dose** | **Week** | **IGA 0/1 (%)** |
| --- | --- | --- | --- | --- |
| ***Head-to-head trials*** | | | | |
| JADE COMPARE | Dupilumab | 300 mg Q2W | 16 | 38.80 |
| JADE COMPARE | Abrocitinib | 200 mg QD | 16 | 47.50 |
| JADE COMPARE | Abrocitinib | 100 mg QD | 16 | 34.80 |
| JADE DARE trial | Abrocitinib | 200 mg QD | 16 | 55.30 |
| JADE DARE trial | Dupilumab | 300 mg Q2W | 16 | 42.50 |
| Heads Up trial | Upadacitinib | 30 mg QD | 16 | NR |
| Heads Up trial | Dupilumab | 300 mg Q2W | 16 | NR |
| ***Abrocitinib*** | | | | |
| Gooderham et al., 2019 | Abrocitinib | 100 mg QD | 12 | 29.60 |
| Gooderham et al., 2019 | Abrocitinib | 200 mg QD | 12 | 43.80 |
| ***Upadacitinib*** | | | | |
| Guttman-Yassky et al., 2020 | Upadacitinib | 15 mg QD | 16 | 43.00 |
| Guttman-Yassky et al., 2020 | Upadacitinib | 30 mg QD | 16 | 50.00 |
| ***Baricitinib*** | | | | |
| BREEZE-AD1 | Baricitinib | 2 mg QD | 16 | 11.40 |
| BREEZE-AD1 | Baricitinib | 4 mg QD | 16 | 16.80 |
| BREEZE-AD2 | Baricitinib | 2 mg QD | 16 | 10.60 |
| BREEZE-AD2 | Baricitinib | 4 mg QD | 16 | 13.80 |
| Guttman-Yassky et al., 2019 | Baricitinib | 2 mg QD | 16 | 22.00 |
| Guttman-Yassky et al., 2019 | Baricitinib | 4 mg QD | 16 | 21.00 |
| BREEZE-AD4 | Baricitinib | 2 mg QD | 16 | 15.00 |
| BREEZE-AD4 | Baricitinib | 4 mg QD | 16 | 22.00 |
| BREEZE-AD7 | Baricitinib | 2 mg QD | 16 | 24.00 |
| BREEZE-AD7 | Baricitinib | 4 mg QD | 16 | 31.00 |
| ***Dupilumab*** | | | | |
| Zhao et al., 2022 | Dupilumab | 300 mg Q2W | 16 | 26.80 |
| LIBERTY AD CHRONOS | Dupilumab | 300 mg Q2W | 16 | 39.00 |
| SOLO 1 | Dupilumab | 300 mg Q2W | 16 | 38.00 |
| SOLO 2 | Dupilumab | 300 mg Q2W | 16 | 36.00 |
| LIBERTY AD CAFÉ | Dupilumab | 300 mg Q2W | 16 | 40.20 |
| ***Tralokinumab*** | | | | |
| ECZTRA 1 | Tralokinumab | 300 mg Q2W | 16 | 15.80 |
| ECZTRA 2 | Tralokinumab | 300 mg Q2W | 16 | 22.20 |
| ECZTRA 3 | Tralokinumab | 300 mg Q2W | 16 | 38.90 |
| ***Lebrikizumab*** | | | | |
| Guttman-Yassky et al., 2020 | Lebrikizumab | 250 mg Q2W | 16 | 44.60 |
| ***Nemolizumab*** | | | | |
| Silverberg 2020 JACI | Nemolizumab | 30 mg Q4W | 16 | 33.3 |

**Supplementary Table S10.** SCORing Atopic Dermatitis (SCORAD) outcomes reported at all tested doses across all included RCTs. Four outcome types are reported where available; mean absolute change from baseline, mean percentage change from baseline, proportion achieving SCORAD-50 (≥50% improvement), and proportion achieving SCORAD-75 (≥75% improvement). QD, once daily, QW, once weekly. Q2W, every 2 weeks. Q4W, every 4 weeks. TCS, topical corticosteroids. NR, not reported.

| **Study ID** | **Drug** | **Dose** | **Week** | **Mean Δ**  **from baseline** | **% Δ**  **from baseline** | **SCORAD-50**  **(%)** | **SCORAD-75**  **(%)** |
| --- | --- | --- | --- | --- | --- | --- | --- |
| JADE COMPARE | Dupilumab | 300 mg Q2W | 16 | NR | NR | NR | NR |
| JADE COMPARE | Abrocitinib | 200 mg QD | 16 | NR | NR | NR | NR |
| JADE COMPARE | Abrocitinib | 100 mg QD | 16 | NR | NR | NR | NR |
| JADE COMPARE | Placebo | — | 16 | NR | NR | NR | NR |
| Zhao et al., 2022 | Dupilumab | 300 mg Q2W | 16 | NR | NR | NR | NR |
| Zhao et al., 2022 | Placebo | — | 16 | NR | NR | NR | NR |
| TREBLE | Lebrikizumab | 125 mg Q4W | 12 | NR | NR | 51.0 | NR |
| TREBLE | Lebrikizumab | 125 mg single dose | 12 | NR | NR | 34.6 | NR |
| TREBLE | Lebrikizumab | 250 mg single dose | 12 | NR | NR | 47.2 | NR |
| TREBLE | Placebo | — | 12 | NR | NR | 26.4 | NR |
| ECZTRA 3 | Tralokinumab | 300 mg Q2W | 16 | -37.7 | NR | NR | NR |
| ECZTRA 3 | Placebo | — | 16 | -26.8 | NR | NR | NR |
| Gooderham et al., 2019 | Abrocitinib | 10 mg QD | 12 | NR | NR | NR | NR |
| Gooderham et al., 2019 | Abrocitinib | 30 mg QD | 12 | NR | NR | NR | NR |
| Gooderham et al., 2019 | Abrocitinib | 100 mg QD | 12 | NR | NR | NR | NR |
| Gooderham et al., 2019 | Abrocitinib | 200 mg QD | 12 | NR | -69.7 | NR | NR |
| Gooderham et al., 2019 | Placebo | — | 12 | NR | -29.0 | NR | NR |
| Guttman-Yassky et al., 2020 | Upadacitinib | 7.5 mg QD | 16 | NR | NR | NR | NR |
| Guttman-Yassky et al., 2020 | Upadacitinib | 15 mg QD | 16 | NR | NR | NR | NR |
| Guttman-Yassky et al., 2020 | Upadacitinib | 30 mg QD | 16 | NR | NR | NR | NR |
| Guttman-Yassky et al., 2020 | Placebo | — | 16 | NR | NR | NR | NR |
| BREEZE-AD7 | Baricitinib | 2 mg QD | 16 | NR | NR | NR | 11.0 |
| BREEZE-AD7 | Baricitinib | 4 mg QD | 16 | NR | NR | NR | 18.0 |
| BREEZE-AD7 | Placebo | — | 16 | NR | NR | NR | 7.0 |
| Thaçi et al., 2016 | Dupilumab | 300 mg QW | 16 | NR | -56.9 | NR | NR |
| Thaçi et al., 2016 | Placebo | — | 16 | NR | -13.8 | NR | NR |
| Guttman-Yassky et al., 2020 | Lebrikizumab | 125 mg Q4W | 16 | NR | NR | NR | NR |
| Guttman-Yassky et al., 2020 | Lebrikizumab | 250 mg Q4W | 16 | NR | NR | NR | NR |
| Guttman-Yassky et al., 2020 | Lebrikizumab | 250 mg Q2W | 16 | NR | NR | NR | NR |
| Guttman-Yassky et al., 2020 | Placebo | — | 16 | NR | NR | NR | NR |
| Ruzicka et al., 2017 | Nemolizumab | 0.1 mg/kg Q4W | 12 | NR | NR | NR | NR |
| Ruzicka et al., 2017 | Nemolizumab | 0.5 mg/kg Q4W | 12 | NR | NR | NR | NR |
| Ruzicka et al., 2017 | Nemolizumab | 2.0 mg/kg Q4W | 12 | -39.8 | NR | NR | 16.2 |
| Ruzicka et al., 2017 | Placebo | — | 12 | -18.5 | NR | NR | 3.1 |
| LIBERTY AD CHRONOS | Dupilumab | 300 mg QW | 16 | NR | -63.3 | NR | NR |
| LIBERTY AD CHRONOS | Dupilumab | 300 mg Q2W | 16 | NR | -62.1 | NR | NR |
| LIBERTY AD CHRONOS | Placebo | — | 16 | NR | -31.8 | NR | NR |
| BREEZE-AD1 | Baricitinib | 1 mg QD | 16 | NR | NR | NR | NR |
| BREEZE-AD1 | Baricitinib | 2 mg QD | 16 | NR | NR | NR | NR |
| BREEZE-AD1 | Baricitinib | 4 mg QD | 16 | NR | NR | NR | 10.4 |
| BREEZE-AD1 | Placebo | — | 16 | NR | NR | NR | 1.2 |
| BREEZE-AD2 | Baricitinib | 1 mg QD | 16 | NR | NR | NR | NR |
| BREEZE-AD2 | Baricitinib | 2 mg QD | 16 | NR | NR | NR | NR |
| BREEZE-AD2 | Baricitinib | 4 mg QD | 16 | NR | NR | NR | 11.4 |
| BREEZE-AD2 | Placebo | — | 16 | NR | NR | NR | 1.6 |
| Heads Up trial | Upadacitinib | 30 mg QD | 16 | NR | NR | NR | NR |
| Heads Up trial | Dupilumab | 300 mg Q2W | 16 | NR | NR | NR | NR |
| JADE DARE trial | Abrocitinib | 200 mg QD | 16 | NR | NR | NR | NR |
| JADE DARE trial | Dupilumab | 300 mg Q2W | 16 | NR | NR | NR | NR |
| Silverberg 2020 JACI | Nemolizumab | 10 mg Q4W | 24 | -34.6 | NR | NR | NR |
| Silverberg 2020 JACI | Nemolizumab | 30 mg Q4W | 24 | -37.8 | NR | NR | NR |
| Silverberg 2020 JACI | Nemolizumab | 90 mg Q4W | 24 | -32.5 | NR | NR | NR |
| Silverberg 2020 JACI | Placebo | — | 24 | -25.0 | NR | NR | NR |
| DUPISTAD | Dupilumab | 300 mg Q2W | 12 | -37.8 | NR | NR | NR |
| DUPISTAD | Placebo | — | 12 | -20.6 | NR | NR | NR |
| Guttman-Yassky et al., 2019 | Baricitinib | 2 mg QD | 16 | NR | NR | NR | NR |
| Guttman-Yassky et al., 2019 | Baricitinib | 4 mg QD | 16 | NR | -47.0 | NR | NR |
| Guttman-Yassky et al., 2019 | Placebo | — | 16 | NR | -21.0 | NR | NR |
| SOLO 1 | Dupilumab | 300 mg Q2W | 16 | NR | -57.7 | NR | NR |
| SOLO 1 | Dupilumab | 300 mg QW | 16 | NR | NR | NR | NR |
| SOLO 1 | Placebo | — | 16 | NR | -29.0 | NR | NR |
| SOLO 2 | Dupilumab | 300 mg Q2W | 16 | NR | -51.1 | NR | NR |
| SOLO 2 | Dupilumab | 300 mg QW | 16 | NR | NR | NR | NR |
| SOLO 2 | Placebo | — | 16 | NR | -19.7 | NR | NR |
| ECZTRA 1 | Tralokinumab | 300 mg Q2W | 16 | -25.2 | NR | NR | NR |
| ECZTRA 1 | Placebo | — | 16 | -14.7 | NR | NR | NR |
| ECZTRA 2 | Tralokinumab | 300 mg Q2W | 16 | -28.1 | NR | NR | NR |
| ECZTRA 2 | Placebo | — | 16 | -14.0 | NR | NR | NR |
| ECZTRA 7 | Tralokinumab | 300 mg Q2W | 16 | -42.7 | NR | NR | NR |
| ECZTRA 7 | Placebo | — | 16 | -34.1 | NR | NR | NR |
| LIBERTY AD CAFÉ | Dupilumab | 300 mg QW + TCS | 16 | NR | NR | 55.5 | NR |
| LIBERTY AD CAFÉ | Dupilumab | 300 mg Q2W + TCS | 16 | NR | NR | 66.4 | NR |
| LIBERTY AD CAFÉ | Placebo | — | 16 | NR | NR | 25.9 | NR |
| BREEZE-AD4 | Baricitinib | 1 mg QD + TCS | 16 | NR | NR | NR | 6.0 |
| BREEZE-AD4 | Baricitinib | 2 mg QD + TCS | 16 | NR | NR | NR | 8.0 |
| BREEZE-AD4 | Baricitinib | 4 mg QD + TCS | 16 | NR | NR | NR | 7.0 |
| BREEZE-AD4 | Placebo + TCS | — | 16 | NR | NR | NR | 1.0 |

**Supplementary Table S11.** Dermatology Life Quality Index (DLQI) outcomes are reported at all tested doses across all included RCTs. Three outcome types are reported where available: continuous change from baseline, proportion achieving a ≥4-point improvement and proportion achieving DLQI 0-1 (no/minimal impact on quality of life). QD, once daily, QW, once weekly. Q2W, every 2 weeks. Q4W, every 4 weeks. TCS, topical corticosteroids. NR, not reported.

| **Study ID** | **Drug** | **Dose** | **Week** | **DLQI Change**  **(from baseline)** | **DLQI ≥4-point**  **improvement (%)** | **DLQI 0–1 (%)** |
| --- | --- | --- | --- | --- | --- | --- |
| JADE COMPARE | Dupilumab | 300 mg Q2W | 16 | NR | 81.80 | NR |
| JADE COMPARE | Abrocitinib | 200 mg QD | 16 | NR | 86.40 | NR |
| JADE COMPARE | Abrocitinib | 100 mg QD | 16 | NR | 74.70 | NR |
| JADE COMPARE | Placebo | — | 16 | NR | 56.50 | NR |
| Zhao et al., 2022 | Dupilumab | 300 mg Q2W | 16 | -10.33 | NR | NR |
| Zhao et al., 2022 | Placebo | — | 16 | -5.06 | NR | NR |
| TREBLE | Lebrikizumab | 125 mg Q4W | 12 | -43.10 | NR | NR |
| TREBLE | Lebrikizumab | 125 mg single dose | 12 | -34.30 | NR | NR |
| TREBLE | Lebrikizumab | 250 mg single dose | 12 | -40.70 | NR | NR |
| TREBLE | Placebo | — | 12 | -33.60 | NR | NR |
| ECZTRA 3 | Tralokinumab | 300 mg Q2W | 16 | -11.70 | 83.50 | NR |
| ECZTRA 3 | Placebo | — | 16 | -8.80 | 65.90 | NR |
| Gooderham et al., 2019 | Abrocitinib | 10 mg QD | 12 | NR | NR | NR |
| Gooderham et al., 2019 | Abrocitinib | 30 mg QD | 12 | NR | NR | NR |
| Gooderham et al., 2019 | Abrocitinib | 100 mg QD | 12 | NR | NR | NR |
| Gooderham et al., 2019 | Abrocitinib | 200 mg QD | 12 | NR | NR | NR |
| Gooderham et al., 2019 | Placebo | — | 12 | NR | NR | NR |
| Guttman-Yassky et al., 2020 | Upadacitinib | 7.5 mg QD | 16 | NR | NR | NR |
| Guttman-Yassky et al., 2020 | Upadacitinib | 15 mg QD | 16 | NR | NR | NR |
| Guttman-Yassky et al., 2020 | Upadacitinib | 30 mg QD | 16 | NR | NR | NR |
| Guttman-Yassky et al., 2020 | Placebo | — | 16 | NR | NR | NR |
| BREEZE-AD7 | Baricitinib | 2 mg QD | 16 | -7.50 | 17.0 | NR |
| BREEZE-AD7 | Baricitinib | 4 mg QD | 16 | -8.90 | 23.0 | NR |
| BREEZE-AD7 | Placebo | — | 16 | -5.60 | 7.00 | NR |
| Thaçi et al., 2016 | Dupilumab | 300 mg QW | 16 | -10.70 | NR | NR |
| Thaçi et al., 2016 | Placebo | — | 16 | -1.40 | NR | NR |
| Guttman-Yassky et al., 2020 | Lebrikizumab | 125 mg Q4W | 16 | -7.90 | NR | NR |
| Guttman-Yassky et al., 2020 | Lebrikizumab | 250 mg Q4W | 16 | -9.20 | NR | NR |
| Guttman-Yassky et al., 2020 | Lebrikizumab | 250 mg Q2W | 16 | -9.70 | NR | NR |
| Guttman-Yassky et al., 2020 | Placebo | — | 16 | -5.90 | NR | NR |
| Ruzicka et al., 2017 | Nemolizumab | 0.1 mg/kg Q4W | 12 | NR | NR | NR |
| Ruzicka et al., 2017 | Nemolizumab | 0.5 mg/kg Q4W | 12 | NR | NR | NR |
| Ruzicka et al., 2017 | Nemolizumab | 2.0 mg/kg Q4W | 12 | NR | NR | NR |
| Ruzicka et al., 2017 | Placebo | — | 12 | NR | NR | NR |
| LIBERTY AD CHRONOS | Dupilumab | 300 mg QW | 16 | -10.50 | 74.0 | NR |
| LIBERTY AD CHRONOS | Dupilumab | 300 mg Q2W | 16 | -9.70 | 81.0 | NR |
| LIBERTY AD CHRONOS | Placebo | — | 16 | -5.30 | 43.0 | NR |
| BREEZE-AD1 | Baricitinib | 1 mg QD | 16 | -4.60 | NR | 7.9 |
| BREEZE-AD1 | Baricitinib | 2 mg QD | 16 | -4.30 | NR | 11.4 |
| BREEZE-AD1 | Baricitinib | 4 mg QD | 16 | -6.80 | NR | 16.8 |
| BREEZE-AD1 | Placebo | — | 16 | -2.50 | NR | 4.8 |
| BREEZE-AD2 | Baricitinib | 1 mg QD | 16 | -5.10 | NR | 9.6 |
| BREEZE-AD2 | Baricitinib | 2 mg QD | 16 | -7.40 | NR | 11.4 |
| BREEZE-AD2 | Baricitinib | 4 mg QD | 16 | -7.60 | NR | 15.4 |
| BREEZE-AD2 | Placebo | — | 16 | -3.40 | NR | 2.9 |
| Heads Up trial | Upadacitinib | 30 mg QD | 16 | NR | NR | NR |
| Heads Up trial | Dupilumab | 300 mg Q2W | 16 | NR | NR | NR |
| JADE DARE trial | Abrocitinib | 200 mg QD | 16 | -10.80 | 82.7 | NR |
| JADE DARE trial | Dupilumab | 300 mg Q2W | 16 | -10.00 | 84.2 | NR |
| Silverberg 2020 JACI | Nemolizumab | 10 mg Q4W | 24 | -11.6 | NR | NR |
| Silverberg 2020 JACI | Nemolizumab | 30 mg Q4W | 24 | -10.5 | NR | NR |
| Silverberg 2020 JACI | Nemolizumab | 90 mg Q4W | 24 | -10.9 | NR | NR |
| Silverberg 2020 JACI | Placebo | — | 24 | -8.6 | NR | NR |
| DUPISTAD | Dupilumab | 300 mg Q2W | 12 | -11.80 | 72.2 | NR |
| DUPISTAD | Placebo | — | 12 | -7.50 | 44.6 | NR |
| Guttman-Yassky et al., 2019 | Baricitinib | 2 mg QD | 16 | -7.50 | NR | NR |
| Guttman-Yassky et al., 2019 | Baricitinib | 4 mg QD | 16 | -8.00 | NR | NR |
| Guttman-Yassky et al., 2019 | Placebo | — | 16 | -5.50 | NR | NR |
| SOLO 1 | Dupilumab | 300 mg Q2W | 16 | -9.30 | NR | NR |
| SOLO 1 | Dupilumab | 300 mg QW | 16 | -9.00 | NR | NR |
| SOLO 1 | Placebo | — | 16 | -5.30 | NR | NR |
| SOLO 2 | Dupilumab | 300 mg Q2W | 16 | -9.30 | NR | NR |
| SOLO 2 | Dupilumab | 300 mg QW | 16 | -9.50 | NR | NR |
| SOLO 2 | Placebo | — | 16 | -3.60 | NR | NR |
| ECZTRA 1 | Tralokinumab | 300 mg Q2W | 16 | -7.10 | 44.6 | NR |
| ECZTRA 1 | Placebo | — | 16 | -5.00 | 31.6 | NR |
| ECZTRA 2 | Tralokinumab | 300 mg Q2W | 16 | -8.80 | 56.3 | NR |
| ECZTRA 2 | Placebo | — | 16 | -4.90 | 27.3 | NR |
| ECZTRA 7 | Tralokinumab | 300 mg Q2W | 16 | -11.20 | NR | NR |
| ECZTRA 7 | Placebo | — | 16 | -9.60 | NR | NR |
| LIBERTY AD CAFÉ | Dupilumab | 300 mg QW + TCS | 16 | -8.80 | 77.8 | NR |
| LIBERTY AD CAFÉ | Dupilumab | 300 mg Q2W + TCS | 16 | -9.50 | 87.6 | NR |
| LIBERTY AD CAFÉ | Placebo | — | 16 | -4.50 | 44.2 | NR |
| BREEZE-AD4 | Baricitinib | 1 mg QD + TCS | 16 | -6.18 | NR | 13.0 |
| BREEZE-AD4 | Baricitinib | 2 mg QD + TCS | 16 | -6.57 | NR | 18.0 |
| BREEZE-AD4 | Baricitinib | 4 mg QD + TCS | 16 | -7.95 | NR | 29.0 |
| BREEZE-AD4 | Placebo + TCS | — | 16 | -4.95 | NR | 10.0 |

**Supplementary Table S12.** Study-level safety outcomes reported at all tested doses across included randomized controlled trials. Any adverse event (AE), serious AE, discontinuation due to AE, and the three most reported AEs (name, n, %). QD, once daily, QW, once weekly. Q2W, every 2 weeks. Q4W, every 4 weeks. TCS, topical corticosteroids. URTI, upper respiratory tract infection. CPK, creatine phosphokinase. UTI, urinary tract infection. D/C, discontinuation

| **Study ID** | **Drug** | **Dose** | **Wk** | **N** | **Any AE**  **n (%)** | **Serious AE**  **n (%)** | **D/C due to AE**  **n (%)** | **AE1** | **AE2** | **AE3** |
| --- | --- | --- | --- | --- | --- | --- | --- | --- | --- | --- |
| JADE COMPARE | Dupilumab | 300 mg Q2W | 16 | 242 | 121 (50.0) | 2 (0.8) | 8 (3.3) | Conjunctivitis, 15 (6.2%) | Headache, 13 (5.4%) | Nausea, 7 (2.9%) |
| JADE COMPARE | Abrocitinib | 200 mg QD | 16 | 226 | 140 (61.9) | 2 (0.9) | 10 (4.4) | Nausea, 25 (11.1%) | Acne, 15 (6.6%) | Headache, 15 (6.6%) |
| JADE COMPARE | Abrocitinib | 100 mg QD | 16 | 238 | 121 (50.8) | 6 (2.5) | 6 (2.5) | Headache, 10 (4.2%) | Nausea, 10 (4.2%) | Acne, 7 (2.9%) |
| JADE COMPARE | Placebo | — | 16 | 131 | 70 (53.4) | 3 (2.3) | 5(3.8) | Headache, 7 (5.3%) | Conjunctivitis, 3 (2.3%) | Nausea, 2 (1.5%) |
| Zhao et al., 2022 | Dupilumab | 300 mg Q2W | 16 | 82 | 63 (77.0) | 1 (1.0) | 4 (5.0) | Atopic Dermatitis, 20 (24%) | URTI, 9 (11%) | Eye disorders (non-conjunctivitis), 9 (11%) |
| Zhao et al., 2022 | Placebo | — | 16 | 83 | 67 (81.0) | 3 (4.0) | 2 (2.0) | Atopic Dermatitis, 38 (46%) | URTI, 6 (7%) | Proteinuria, 7 (8%) |
| TREBLE | Lebrikizumab | 125 mg Q4W | 12 | 50 | 27 (54.0) | 2 (4.0) | 2 (4.0) | Skin infections, 3 (6%) | Conjunctivitis, 3 (6%) | Local injection site reactions, 2 (4%) |
| TREBLE | Lebrikizumab | 125 mg single dose | 12 | 54 | 38 (70.0) | 3 (6.0) | 1 (2.0) | Conjunctivitis, 7 (13%) | Skin infections, 6 (11%) | Allergic conjunctivitis, 4 (7%) |
| TREBLE | Lebrikizumab | 250 mg single dose | 12 | 52 | 39 (75.0) | 0 (0.0) | 0 (0.0) | Conjunctivitis, 5 (10%) | Skin infections, 5 (10%) | Herpes simplex, 2 (4%) |
| TREBLE | Placebo | — | 12 | 53 | 35 (66.0) | 2 (4.0) | 1 (2.0) | Skin infections, 9 (17%) | Conjunctivitis, 4 (8%) | Local injection site reactions, 1 (2%) |
| ECZTRA 3 | Tralokinumab | 300 mg Q2W | 16 | 252 | 180 (71.4) | 2 (0.8) | 6 (2.4) | Viral URTI, 49 (19.4%) | Conjunctivitis, 28 (11.1%) | Headache, 22 (8.7%) |
| ECZTRA 3 | Placebo | — | 16 | 126 | 84 (66.7) | 4 (3.2) | 1 (0.8) | Viral URTI, 14 (11.1%) | Atopic Dermatitis, 10 (7.9%) | Headache, 6 (4.8%) |
| Gooderham et al., 2019 | Abrocitinib | 10 mg QD | 12 | 49 | 23 (46.9) | 2 (4.1) | 8 (16.3%) | Atopic Dermatitis, 8 (16.3%) | Viral URTI, 5 (10.2%) | Diarrhea, 3 (6.1%) |
| Gooderham et al., 2019 | Abrocitinib | 30 mg QD | 12 | 51 | 19 (37.3) | 0 (0.0) | 8 (15.7%) | Atopic Dermatitis, 9 (17.6%) | Viral URTI, 6 (11.8%) | Headache, 5 (9.8%) |
| Gooderham et al., 2019 | Abrocitinib | 100 mg QD | 12 | 56 | 12 (21.4%) | 3 (5.4) | 3 (5.4) | Viral URTI, 10 (17.9%) | Atopic Dermatitis, 7 (12.5%) | Headache, 5 (8.9%) |
| Gooderham et al., 2019 | Abrocitinib | 200 mg QD | 12 | 55 | 8 (14.5%) | 2 (3.6) | 1 (1.8) | Diarrhea, 9 (16.4%) | Viral URTI, 7 (12.7%) | Atopic Dermatitis, 7 (12.7%) |
| Gooderham et al., 2019 | Placebo | — | 12 | 56 | 9 (16.1%) | 2 (3.6) | 4 (7.1) | Atopic Dermatitis, 7 (12.5%) | Viral URTI, 5 (8.9%) | URTI, 5 (8.9%) |
| Guttman-Yassky et al., 2020 | Upadacitinib | 7.5 mg QD | 16 | 42 | 31 (74.0) | 2 (4.8) | 4 (9.5) | URTI, 7 (17%) | Atopic Dermatitis, 6 (14%) | Acne, 4 (9.5%) |
| Guttman-Yassky et al., 2020 | Upadacitinib | 15 mg QD | 16 | 42 | 32 (76.0) | 1 (2.4) | 2 (4.8) | URTI, 5 (12%) | Headache, Nasopharyngitis, CPK Elevation, 3 (7.1%) | Acne, Atopic Dermatitis, Diarrhea, Neutropenia, Hepatic AEs, 2 (4.8%) |
| Guttman-Yassky et al., 2020 | Upadacitinib | 30 mg QD | 16 | 42 | 33 (79.0) | 0 (0.0) | 4 (9.5) | Acne, 6 (14.3%) | URTI, 5 (12%) | Atopic Dermatitis, Headache, CPK Elevation, 4 (9.5%) |
| Guttman-Yassky et al., 2020 | Placebo | — | 16 | 40 | 25 (63.0) | 1 (2.5) | 3 (7.5) | URTI, 4 (10%) | Atopic Dermatitis, Diarrhea, Contact Dermatitis, CPK Elevation, Hematuria, Proteinuria, Ligament Sprain, 2 (5%) | Acne, Headache, Nasopharyngitis, Hepatic disorders, 1 (2.5%) |
| BREEZE-AD7 | Baricitinib | 2 mg QD | 16 | 109 | 61 (56.0) | 2 (2.0) | 0 (0.0) | Nasopharyngitis, 12 (11%) | URTI, 8 (7%) | Folliculitis, 7 (6%) |
| BREEZE-AD7 | Baricitinib | 4 mg QD | 16 | 111 | 64 (58.0) | 4 (4.0) | 5 (5.0) | Nasopharyngitis, 17 (15%) | Folliculitis, 10 (9%) | Herpes simplex, 7 (7%) |
| BREEZE-AD7 | Placebo | — | 16 | 108 | 41 (38.0) | 4 (4.0) | 1 (1.0) | Nasopharyngitis, 13 (12%) | Herpes simplex, Allergic rhinitis, Oropharyngeal pain, 3 (3%) | URTI, Conjunctivitis, Folliculitis, Back pain, 2 (2%) |
| Thaçi et al., 2016 | Dupilumab | 300 mg QW | 16 | 63 | 53 (84.0) | 1 (2.0) | 1 (2.0) | Nasopharyngitis, 16 (25%) | Atopic Dermatitis, 8 (13%) | Headache, 8 (13%) |
| Thaçi et al., 2016 | Placebo | — | 16 | 61 | 49 (80.0) | 4 (7.0) | 3 (5.0) | Nasopharyngitis, 16 (26%) | URTI, Atopic Dermatitis, 11 (18%) | Back Pain, 5 (8%) |
| Guttman-Yassky et al., 2020 | Lebrikizumab | 125 mg Q4W | 16 | 73 | 42 (57.5) | 2 (2.7) | 2 (2.7) | URTI, 6 (8.2%) | Nasopharyngitis, 4 (5.5%) | Herpesvirus infections, Headache, Injection Site Reactions, 2 (2.7%) |
| Guttman-Yassky et al., 2020 | Lebrikizumab | 250 mg Q4W | 16 | 80 | 39 (48.8) | 0 (0.0) | 4 (5.0) | URTI, 9 (11.3%) | Herpesvirus infections, 4 (5%) | Fatigue, 4 (5%) |
| Guttman-Yassky et al., 2020 | Lebrikizumab | 250 mg Q2W | 16 | 75 | 46 (61.3) | 2 (2.7) | 3 (4.0) | Nasopharyngitis, 9 (12%) | Injection site reactions, 7 (9.3%) | Headache, Injection Site Pain, 4 (5.3%) |
| Guttman-Yassky et al., 2020 | Placebo | — | 16 | 52 | 24 (46.2) | 2 (3.8) | 1 (1.9) | URTI, 3 (5.8%) | Headache, 3 (5.8%) | Herpesvirus infections, Nasopharyngitis, 2 (3.8%) |
| Ruzicka et al., 2017 | Nemolizumab | 0.1 mg/kg Q4W | 12 | 53 | 38 (72.0) | 1 (2.0) | 5 (9.0) | Atopic Dermatitis, 11 (21%) | Nasopharyngitis, 9 (17%) | Conjunctivitis, 4 (8%) |
| Ruzicka et al., 2017 | Nemolizumab | 0.5 mg/kg Q4W | 12 | 54 | 36 (67.0) | 0 (0.0) | 3 (6.0) | Atopic Dermatitis, 10 (19%) | Nasopharyngitis, 6 (11%) | Peripheral Edema, 3 (6%) |
| Ruzicka et al., 2017 | Nemolizumab | 2.0 mg/kg Q4W | 12 | 52 | 40 (77.0) | 1 (2.0) | 4 (8.0) | Atopic Dermatitis, 11 (21%) | Nasopharyngitis, 5 (10%) | URTI, Peripheral Edema, 5 (10%) |
| Ruzicka et al., 2017 | Placebo | — | 12 | 53 | 36 (68.0) | 3 (6.0) | 1 (2.0) | Nasopharyngitis, 8 (15%) | Atopic Dermatitis, 7 (13%) | URTI, 6 (11%) |
| LIBERTY AD CHRONOS | Dupilumab | 300 mg QW | 16 | 315 | 261 (83.0) | 9 (3.0) | 9 (3.0) | Conjunctivitis, 61 (19%) | Nasopharyngitis, 60 (19%) | Injection Site Reactions, 60 (19%) |
| LIBERTY AD CHRONOS | Dupilumab | 300 mg Q2W | 16 | 110 | 97 (88.0) | 4 (4.0) | 2 (2.0) | Nasopharyngitis, 25 (23%) | Atopic Dermatitis, 20 (18%) | Injection Site Reactions, 16 (15%) |
| LIBERTY AD CHRONOS | Placebo | — | 16 | 315 | 266 (84.0) | 16 (5.0) | 24 (8.0) | Atopic Dermatitis, 144 (46%) | Nasopharyngitis, 61 (19%) | URTI, 32 (10%) |
| BREEZE-AD1 | Baricitinib | 1 mg QD | 16 | 127 | 69 (54.3) | 1 (0.8) | 2 (1.6%) | Nasopharyngitis, 22 (17.3%) | Diarrhoea, 9 (7.1%) | Herpes simplex, 7 (5.5%) |
| BREEZE-AD1 | Baricitinib | 2 mg QD | 16 | 123 | 71 (57.7) | 0 (0.0) | 1 (0.8%) | Headache, 14 (11.4%) | Nasopharyngitis, 12 (9.8%) | Skin Infections Requiring Antibiotics, 6 (4.9%) |
| BREEZE-AD1 | Baricitinib | 4 mg QD | 16 | 125 | 73 (58.4) | 2 (1.6) | 1 (0.8) | Nasopharyngitis, 12 (9.6%) | Headache, 10 (8%) | Herpes simplex, 9 (7.2%) |
| BREEZE-AD1 | Placebo | — | 16 | 249 | 135 (54.2) | 6 (2.4) | 4 (1.6) | Nasopharyngitis, 26 (10.4%) | Headache, 16 (6.4%) | Skin infections requiring antibiotics, 11 (4.4%) |
| BREEZE-AD2 | Baricitinib | 1 mg QD | 16 | 124 | 66 (53.2) | 9 (7.3) | 7 (5.6) | Nasopharyngitis, 13 (10.5%) | Herpes Simplex, 6 (4.8%) | Conjunctivitis/Keratitis, URTI, 6 (4.8%) |
| BREEZE-AD2 | Baricitinib | 2 mg QD | 16 | 123 | 71 (57.7) | 3 (2.4) | 3 (2.4) | Nasopharyngitis, 16 (13%) | Headache, Skin Infections Requiring Antibiotics, 9 (7.3%) | Herpes Simplex, 7 (5.7%) |
| BREEZE-AD2 | Baricitinib | 4 mg QD | 16 | 123 | 66 (53.7) | 1 (0.8) | 2 (1.6) | Headache, 11 (8.9%) | Nasopharyngitis, 10 (8.1%) | CPK Elevation, 7 (5.7%) |
| BREEZE-AD2 | Placebo | — | 16 | 244 | 137 (56.1) | 9 (3.7) | 2 (0.8) | Nasopharyngitis, 30 (12.3%) | Skin Infections Requiring Antibiotics, 19 (7.8%) | Herpes Simplex, 11 (4.5%) |
| Heads Up trial | Upadacitinib | 30 mg QD | 16 | 342 | 249 (72.8) | 10 (2.9) | 7 (2.0) | Acne, 55 (16.1%) | Atopic Dermatitis, 24 (7%) | CPK Elevation, 23 (6.7%) |
| Heads Up trial | Dupilumab | 300 mg Q2W | 16 | 331 | 215 (65.0) | 4 (1.2) | 4 (1.2) | Conjunctivitis, 29 (8.8%) | Atopic Dermatitis, 29 (8.8%) | Nasopharyngitis, 22 (6.6%) |
| JADE DARE trial | Abrocitinib | 200 mg QD | 16 | 362 | 268 (74.0) | 6 (2.0) | 12 (3.0) | Nausea, 70 (19%) | Acne or Folliculitis, 48 (13%) | Headache, 47 (13%) |
| JADE DARE trial | Dupilumab | 300 mg Q2W | 16 | 365 | 239 (65.0) | 6 (2.0) | 9 (2.0) | Conjunctivitis, 39 (11%) | Headache, 24 (7%) | Herpes Simplex, 18 (5%) |
| Silverberg 2020 JACI | Nemolizumab | 30 mg Q4W | 16 | 57 | 47 (82.5) | 2 (3.5) | 2 (3.5) | Nasopharyngitis, 14 (24.6%) | Atopic Dermatitis, 14 (24.6%) | Asthma Events, 7 (12.3%) |
| Silverberg 2020 JACI | Placebo | — | 16 | 56 | 43 (76.8) | 1 (1.8) | 4 (7.1) | Atopic Dermatitis, 18 (32.1%) | Nasopharyngitis, 12 (21.4%) | Herpes Infections, 5 (8.9%) |
| DUPISTAD | Dupilumab | 300 mg Q2W | 12 | 127 | 72 (56.7) | 2 (1.6) | 3 (2.4) | Conjunctivitis, 12 (9.4%) | Headache, 9 (7.1%) | Dry eyes, 5 (3.9%) |
| DUPISTAD | Placebo | — | 12 | 61 | 41 (67.2) | 1 (1.6) | 1 (1.6) | Atopic Dermatitis, 8 (13.1%) | Headache, 5 (8.2%) | Oral herpes, Injection site reactions, 3 (4.9%) |
| Guttman-Yassky et al., 2019 | Baricitinib | 2 mg QD | 16 | 37 | 17 (46.0) | 0 (0.0) | 1 (3.0) | Headache, 2 (5%) | UTI, 1 (3%) | CPK Elevation, 1 (3%) |
| Guttman-Yassky et al., 2019 | Baricitinib | 4 mg QD | 16 | 38 | 27 (71.0) | 1 (3.0) | 5 (13.0) | Headache, 5 (13%) | CPK Elevation, 5 (13%) | URTI, leukopenia/neutropenia, 2 (5%) |
| Guttman-Yassky et al., 2019 | Placebo | — | 16 | 49 | 24 (49.0) | 0 (0.0) | 5 (10.0) | Atopic Dermatitis, 4 (8%) | Lymphopenia, 3 (6%) | URTI, 1 (2%) |
| SOLO 1 | Dupilumab | 300 mg Q2W | 16 | 229 | 167 (73.0) | 7 (3.0) | 4 (2.0) | Headache, 21 (9%) | Injection Site Reactions, 19 (8%) | Herpes Viral Infections, 15 (7%) |
| SOLO 1 | Dupilumab | 300 mg QW | 16 | 218 | 150 (69.0) | 2 (1.0) | 4 (2.0) | Injection Site Reactions, 41 (19%) | Adjudicated Skin Infection, 14 (6%) | URTI, 11 (5%) |
| SOLO 1 | Placebo | — | 16 | 222 | 145 (65.0) | 11 (5.0) | 2 (1.0) | Adjudicated Skin Infection, 18 (8%) | Injection Site Reactions, 13 (6%) | Headache, 13 (6%) |
| SOLO 2 | Dupilumab | 300 mg Q2W | 16 | 236 | 154 (65.0) | 4 (2.0) | 2 (1.0) | Injection Site Reactions, 32 (14%) | Headache, 19 (8%) | Herpes Viral Infections, 10 (4%) |
| SOLO 2 | Dupilumab | 300 mg QW | 16 | 237 | 157 (66.0) | 8 (3.0) | 3 (1.0) | Injection Site Reactions, 31 (13%) | Headache, 22 (9%) | Herpes Viral Infections, 12 (5%) |
| SOLO 2 | Placebo | — | 16 | 234 | 168 (72.0) | 13 (6.0) | 5 (2.0) | Adjudicated Skin Infection, 26 (11%) | Injection Site Reactions, 15 (6%) | Headache, 11 (5%) |
| ECZTRA 1 | Tralokinumab | 300 mg Q2W | 16 | 601 | 460 (76.4) | 23 (3.8) | 20 (3.3) | Atopic Dermatitis, 156 (25.9%) | Viral URTI, 139 (23.1%) | Conjunctivitis, 43 (7.1%) |
| ECZTRA 1 | Placebo | — | 16 | 197 | 151 (77.0) | 8 (4.1) | 8 (4.1) | Atopic Dermatitis, 75 (38.3%) | Viral URTI, 41 (20.9%) | Headache, 10 (5.1%) |
| ECZTRA 2 | Tralokinumab | 300 mg Q2W | 16 | 591 | 364 (61.5) | 10 (1.7) | 9 (1.5) | Atopic Dermatitis, 98 (16.6%) | Viral URTI, 49 (8.3%) | Conjunctivitis, 18 (3%) |
| ECZTRA 2 | Placebo | — | 16 | 201 | 132 (66.0) | 3 (2.5) | 3 (1.5) | Atopic Dermatitis, 67 (33.5%) | Skin Infections Requiring Systemic Treatment, 22 (11%) | Viral URTI, 17 (8.5%) |
| ECZTRA 7 | Tralokinumab | 300 mg Q2W | 16 | 138 | 107 (77.5) | 1 (0.7) | 1 (0.7) | Viral URTI, 37 (26.8%) | Headache, 21 (15.2%) | Conjunctivitis, 13 (9.4%) |
| ECZTRA 7 | Placebo | — | 16 | 137 | 108 (78.8) | 5 (3.6) | 3 (2.2) | Viral URTI, 35 (25.5%) | Atopic Dermatitis, 16 (11.7%) | Headache, 13 (9.5%) |
| LIBERTY AD CAFÉ | Dupilumab | 300 mg QW + TCS | 16 | 110 | 76 (69.1) | 2 (1.8) | 2 (1.8) | TE-Conjunctivitis, 18 (16.4%) | Nasopharyngitis, 17 (15.5%) | Allergic conjunctivitis, 10 (9.1%) |
| LIBERTY AD CAFÉ | Dupilumab | 300 mg Q2W + TCS | 16 | 107 | 77 (72.0) | 2 (1.9) | 0 (0.0) | TE-Conjunctivitis, 30 (28%) | Nasopharyngitis, 22 (20.6%) | Allergic conjunctivitis, 16 (15%) |
| LIBERTY AD CAFÉ | Placebo | — | 16 | 108 | 75 (69.4) | 2 (1.9) | 1 (0.9) | Nasopharyngitis, 18 (16.7%) | Atopic Dermatitis, 16 (14.8%) | TE-Conjunctivitis, 12 (11.1%) |
| BREEZE-AD4 | Baricitinib | 1 mg QD + TCS | 16 | 93 | 68 (73.1) | 7 (7.5) | 1 (1.1) | CPK Elevation, 21 (22.8%) | Nasopharyngitis, 16 (17.2%) | Skin Infections requiring antibiotics, 12 (12.9%) |
| BREEZE-AD4 | Baricitinib | 2 mg QD + TCS | 16 | 185 | 149 (81.0) | 9 (4.9) | 10 (5.4) | CPK Elevation, 47 (25.7%) | Nasopharyngitis, 38 (20.7%) | Skin Infections requiring antibiotics, 24 (13%) |
| BREEZE-AD4 | Baricitinib | 4 mg QD + TCS | 16 | 92 | 82 (89.1) | 10 (10.9) | 3 (3.3) | Nasopharyngitis, 34 (37%) | CPK Elevation, 27 (29.3%) | Herpes Simplex, 14 (15.2%) |
| BREEZE-AD4 | Placebo + TCS | — | 16 | 93 | 62 (66.7) | 5 (5.4) | 2 (2.2) | CPK Elevation, 16 (17.6%) | Nasopharyngitis, 14 (15.1%) | Headache, Skin Infections Requiring Antibiotics, 8 (8.6%) |

**Supplementary Table S13.** Treatment discontinuation and adverse event (AE)-related discontinuation at all tested doses across all included trials. QW, once weekly. Q2W, every 2 weeks. Q4W, every 4 weeks. LTFU, lost to follow-up. D/C discontinuation. NR, not reported.

| **Study ID** | **Drug** | **Dose** | **Week** | **Discontinued**  **n/N (%)** | **D/C due to AE**  **n/N (%)** | **Main reasons (brief)** |
| --- | --- | --- | --- | --- | --- | --- |
| JADE COMPARE | Abrocitinib | 200 mg QD | 16 | 18/226 (8.0) | 10/226 (4.4) | Withdrawal, AE, protocol, LTFU |
| JADE COMPARE | Abrocitinib | 100 mg QD | 16 | 21/238 (8.8) | 6/238 (2.5) | Withdrawal, AE, protocol, LTFU |
| JADE COMPARE | Dupilumab | 300 mg Q2W | 16 | 19/242 (7.9) | 8/242 (3.3) | Withdrawal, AE, protocol, LTFU |
| JADE COMPARE | Placebo | — | 16 | 14/131 (10.7) | 5/131 (3.8) | Withdrawal, AE, protocol, LTFU |
| Zhao et al., 2022 | Dupilumab | 300 mg Q2W | 16 | 6/82 (7.3) | 4/82 (4.9) | AE, withdrawal, lack of efficacy |
| Zhao et al., 2022 | Placebo | — | 16 | 17/83 (20.5) | 2/83 (2.4%) | Withdrawal, lack of efficacy, AE |
| TREBLE | Placebo | — | 12 | 4/53 (7.5) | 1/53 (2.0) | Withdrawal, LTFU, AE |
| TREBLE | Lebrikizumab | 125 mg Q4W | 12 | 4/51 (7.8) | 2/50 (4.0) | Withdrawal, AE, lack of efficacy |
| TREBLE | Lebrikizumab | 250 mg single dose | 12 | 0/53 (0.0) | 0/52 (0.0) | NR |
| TREBLE | Lebrikizumab | 125 mg single dose | 12 | 3/52 (5.8) | 1/54 (2) | Withdrawal, AE |
| ECZTRA 3 | Tralokinumab | 300 mg Q2W | 16 | 17/253 (6.7) | 6/253 (2.4) | Withdrawal/other, AE, LTFU |
| ECZTRA 3 | Placebo | — | 16 | 6/125 (4.8) | 1/125 (0.8) | Withdrawal/other, AE, LTFU |
| Gooderham et al., 2019 | Placebo | — | 12 | 28/56 (50.0) | 9/56 (16.1) | AE, lack of efficacy, withdrawal, other |
| Gooderham et al., 2019 | Abrocitinib | 10 mg QD | 12 | 22/49 (44.9) | 8/49 (16.3) | AE, lack of efficacy, withdrawal, other |
| Gooderham et al., 2019 | Abrocitinib | 30 mg QD | 12 | 24/51 (47.1) | 8/51 (15.7) | AE, lack of efficacy, withdrawal, other |
| Gooderham et al., 2019 | Abrocitinib | 100 mg QD | 12 | 19/56 (33.9) | 12/56 (21.4) | AE, lack of efficacy, withdrawal, other |
| Gooderham et al., 2019 | Abrocitinib | 200 mg QD | 12 | 17/55 (30.9) | 8/55 (14.5) | AE, lack of efficacy, withdrawal, other |
| Guttman-Yassky et al., 2020 | Placebo | — | 16 | 20/40 (50.0) | 3/40 (7.5) | Lack of efficacy, AE, withdrawal, LTFU |
| Guttman-Yassky et al., 2020 | Upadacitinib | 7.5 mg QD | 16 | 11/42 (26.2) | 4/42 (9.5) | Lack of efficacy, AE, withdrawal, LTFU |
| Guttman-Yassky et al., 2020 | Upadacitinib | 15 mg QD | 16 | 5/42 (11.9) | 2/42 (4.8) | Lack of efficacy, AE, withdrawal, LTFU |
| Guttman-Yassky et al., 2020 | Upadacitinib | 30 mg QD | 16 | 4/42 (9.5) | 4/42 (9.5) | Lack of efficacy, AE, withdrawal, LTFU |
| BREEZE-AD7 | Placebo | — | 16 | 7/109 (6.4) | 1/108 (1.0) | Withdrawal, lack of efficacy, AE |
| BREEZE-AD7 | Baricitinib | 2 mg QD | 16 | 9/109 (8.3) | 0/109 (0.0) | Withdrawal, lack of efficacy, AE |
| BREEZE-AD7 | Baricitinib | 4 mg QD | 16 | 4/111 (3.6) | 5/111 (5.0) | Withdrawal, AE |
| Thaçi et al., 2016 | Placebo | — | 16 | 8/61 (13.1) | 3/61 (5%) | AE, lack of efficacy, withdrawal, LTFU, protocol |
| Thaçi et al., 2016 | Dupilumab | 300 mg QW | 16 | 25/318 (7.9) | 1/63 (2%) | AE, lack of efficacy, withdrawal, LTFU, protocol |
| Guttman-Yassky et al., 2020 | Placebo | — | 16 | 29/52 (55.8) | 1/52 (1.9) | Withdrawal, AE, LTFU, other |
| Guttman-Yassky et al., 2020 | Lebrikizumab | 125 mg Q4W | 16 | 15/73 (20.5) | 2/73 (2.7) | Withdrawal, AE, LTFU, other |
| Guttman-Yassky et al., 2020 | Lebrikizumab | 250 mg Q4W | 16 | 18/80 (22.5) | 4/80 (5.0) | Withdrawal, AE, LTFU, other |
| Guttman-Yassky et al., 2020 | Lebrikizumab | 250 mg Q2W | 16 | 17/75 (22.7) | 3/75 (4.0) | Withdrawal, AE, LTFU, other |
| Ruzicka et al., 2017 | Placebo | — | 12 | 9/53 (17.0) | 1/53 (2.0) | Withdrawal, AE, lack of efficacy |
| Ruzicka et al., 2017 | Nemolizumab | 0.1 mg/kg Q4W | 12 | 9/53 (17.0) | 5/53 (9.0) | Withdrawal, AE, lack of efficacy |
| Ruzicka et al., 2017 | Nemolizumab | 0.5 mg/kg Q4W | 12 | 9/54 (16.7) | 3/54 (6.0) | Withdrawal, AE, lack of efficacy |
| Ruzicka et al., 2017 | Nemolizumab | 2.0 mg/kg Q4W | 12 | 7/52 (13.5) | 4/52 (8.0) | Withdrawal, AE, lack of efficacy |
| LIBERTY AD CHRONOS | Dupilumab | 300 mg QW | 16 | 18/319 (5.6) | 9/315 (3%)* | AE, other |
| LIBERTY AD CHRONOS | Dupilumab | 300 mg Q2W | 16 | 7/106 (6.6) | 2/110 (2%)* | NR |
| LIBERTY AD CHRONOS | Placebo | — | 16 | 33/315 (10.5) | 24/315 (8%)* | Lack of efficacy, AE, withdrawal |
| LIBERTY AD CAFÉ | Placebo | — | 16 | 5/108 (4.6) | 1/108 (0.9) | Lack of efficacy, AE |
| LIBERTY AD CAFÉ | Dupilumab | 300 mg Q2W | 16 | 0/107 (0.0) | 0/107 (0.0) | NR |
| LIBERTY AD CAFÉ | Dupilumab | 300 mg QW | 16 | 2/110 (1.8) | 2/110 (1.8) | AE, physician decision |
| BREEZE-AD1 | Placebo | — | 16 | 23/249 (9.2) | 4/249 (1.6) | Withdrawal, lack of efficacy |
| BREEZE-AD1 | Baricitinib | 1 mg QD | 16 | 11/127 (8.7) | 2/127 (1.6) | Withdrawal, lack of efficacy |
| BREEZE-AD1 | Baricitinib | 2 mg QD | 16 | 10/123 (8.1) | 1/123 (0.8) | Withdrawal, lack of efficacy |
| BREEZE-AD1 | Baricitinib | 4 mg QD | 16 | 5/125 (4.0) | 1/125 (0.8) | Withdrawal, lack of efficacy |
| BREEZE-AD2 | Placebo | — | 16 | 19/244 (7.8) | 2/244 (0.8) | Lack of efficacy, AE, withdrawal |
| BREEZE-AD2 | Baricitinib | 1 mg QD | 16 | 10/125 (8.0) | 7/125 (5.6) | Lack of efficacy, AE, withdrawal |
| BREEZE-AD2 | Baricitinib | 2 mg QD | 16 | 10/123 (8.1) | 3/123 (2.4) | Lack of efficacy, AE, withdrawal |
| BREEZE-AD2 | Baricitinib | 4 mg QD | 16 | 6/123 (4.9) | 2/123 (1.6) | Lack of efficacy, AE |
| Heads Up trial | Upadacitinib | 30 mg QD | 16 | 12/342 (3.5%) | 7/342 (2.0%) | Withdrawal, AE, LTFU |
| Heads Up trial | Dupilumab | 300 mg Q2W | 16 | 15/331 (4.5%) | 4/331 (1.2%) | Withdrawal, AE, LTFU |
| JADE DARE trial | Abrocitinib | 200 mg QD | 16 | 35/362 (9.7) | 12/362 (3.0) | Withdrawal, AE, protocol, LTFU |
| JADE DARE trial | Dupilumab | 300 mg Q2W | 16 | 31/365 (8.5) | 9/365 (2.0) | Withdrawal, AE, protocol, LTFU |
| Silverberg 2020 JACI | Placebo | — | 16 | 14/57 (24.6) | 4/57 (7.1) | Withdrawal, AE, LTFU |
| Silverberg 2020 JACI | Nemolizumab | 10 mg Q4W | 16 | 12/55 (21.8) | 4/55 (7.3) | Withdrawal, AE, LTFU |
| Silverberg 2020 JACI | Nemolizumab | 30 mg Q4W | 16 | 6/57 (10.5) | 2/57 (3.6) | Withdrawal, AE, LTFU |
| Silverberg 2020 JACI | Nemolizumab | 90 mg Q4W | 16 | 13/57 (22.8) | 7/57 (12.3) | Withdrawal, AE, LTFU |
| DUPISTAD | Dupilumab | 300 mg Q2W | 12 | 6/127 (4.7) | 3/127 (2.4) | AE, withdrawal, lack of efficacy |
| DUPISTAD | Placebo | — | 12 | 1/61 (1.6) | 1/61 (1.6) | AE |
| Guttman-Yassky et al., 2019 | Placebo | — | 16 | 20/49 (40.8) | 5/49 (10.0) | Lack of efficacy, AE, withdrawal, LTFU |
| Guttman-Yassky et al., 2019 | Baricitinib | 2 mg QD | 16 | 10/37 (27.0) | 1/37 (3.0) | Lack of efficacy, AE, withdrawal, LTFU |
| Guttman-Yassky et al., 2019 | Baricitinib | 4 mg QD | 16 | 9/38 (23.7) | 5/38 (13.0) | Lack of efficacy, AE, withdrawal, LTFU |
| SOLO 1 | Placebo | — | 16 | 40/224 (17.9) | 2/222 (1) | AE, lack of efficacy, other |
| SOLO 1 | Dupilumab | 300 mg Q2W | 16 | 16/224 (7.1) | 4/229 (2%) | AE, other |
| SOLO 1 | Dupilumab | 300 mg QW | 16 | 26/223 (11.7) | 4/218 (2%) | AE, other |
| SOLO 2 | Placebo | — | 16 | 46/236 (19.5) | 5/234 (2%) | AE, lack of efficacy, other |
| SOLO 2 | Dupilumab | 300 mg Q2W | 16 | 13/233 (5.6) | 2/236 (1%) | AE, other |
| SOLO 2 | Dupilumab | 300 mg QW | 16 | 18/239 (7.5) | 3/237 (1%) | AE, other |
| ECZTRA 1 | Tralokinumab | 300 mg Q2W | 16 | 51/601 (8.5) | 20/601 (3.3) | AE, withdrawal, protocol, LTFU |
| ECZTRA 1 | Placebo | — | 16 | 18/197 (9.1) | 8/197 (4.1) | AE, withdrawal, protocol, LTFU |
| ECZTRA 2 | Tralokinumab | 300 mg Q2W | 16 | 33/591 (5.6) | 9/591 (1.5) | AE, withdrawal, protocol, LTFU |
| ECZTRA 2 | Placebo | — | 16 | 22/201 (11.0) | 3/201 (1.5) | AE, withdrawal, protocol, LTFU |
| ECZTRA 7 | Tralokinumab | 300 mg Q2W | 16 | 13/140 (9.3) | 1/140 (0.7) | AE, other (withdrawal, lack of efficacy, protocol) |
| ECZTRA 7 | Placebo | — | 16 | 17/137 (12.4) | 3/137 (2.2) | AE, other (withdrawal, lack of efficacy, protocol) |
| BREEZE-AD4 | Placebo | — | 16 | 40/93 (43.0) | 2/93 (2.2) | Lack of efficacy, withdrawal, AE |
| BREEZE-AD4 | Baricitinib | 1 mg QD | 16 | 32/93 (34.4) | 1/93 (1.1) | Lack of efficacy, withdrawal, AE |
| BREEZE-AD4 | Baricitinib | 2 mg QD | 16 | 59/185 (31.9) | 10/185 (5.4) | Lack of efficacy, withdrawal, AE |
| BREEZE-AD4 | Baricitinib | 4 mg QD | 16 | 27/92 (29.3) | 3/92 (3.3) | Lack of efficacy, withdrawal, AE |

*: These AE-discontinuation numbers cover the full 52 weeks.

**Supplementary Table S14.** Reasons for treatment discontinuation reported at the study level (pooled across treatment arms unless otherwise noted) across included trials. Discontinuation reasons in this table are drawn from each trial's patient disposition/CONSORT diagram where available. AE, adverse event. LTFU, lost to follow-up. NR, not reported. Reporting categories and the degree of stratification by treatment arm varied across trials, as noted individually.

| **Study ID** | **Drug(s)** | **AE-related**  **n (%)** | **Lack of**  **efficacy n (%)** | **Withdrawal /**  **consent n (%)** | **Protocol /**  **administrative** | **Other /**  **unspecified** | **Notes** |
| --- | --- | --- | --- | --- | --- | --- | --- |
| JADE COMPARE | Abrocitinib, Dupilumab, Placebo | 24 (2.9%) | 2 (0.2%) | 23 (2.7%) | Protocol deviation: 7 (0.8%). Medication error: 2 (0.2%) | LTFU: 6 (0.7%). Pregnancy: 2 (0.2%). Other: 6 (0.7%) | Reasons pooled across arms, from CONSORT diagram, totals reported |
| Zhao et al., 2022 | Dupilumab, Placebo | 6 (3.6%) | 5 (3.0%) (Dupilumab 1 [1.2%], Placebo 4 [4.8%]) | 12 (7.3%) (Dupilumab 1 [1.2%], Placebo 11 [13.3%]) | NR | NR | Reasons reported separately by arm, from CONSORT diagram |
| TREBLE | Lebrikizumab, Placebo | 4 (1.9%) | 1 (0.5%) (Lebrikizumab 1 [0.5%]) | 3 (1.9%) (Placebo 1 [1.9%], Lebrikizumab 2 [1.4%]) | NR | LTFU: 2 (1.0%) (Placebo 2 [3.8%]) | Reasons reported by arm, pooled |
| ECZTRA 3 | Tralokinumab, Placebo | 7 (1.9%) | NR | Included in "other" reasons | Included in "other" reasons | 16 (4.3%) (pooled: includes withdrawal, LTFU, protocol deviations) | Reasons largely pooled across arms, individual categories not fully disaggregated, from CONSORT diagram |
| Gooderham et al., 2019 | Abrocitinib, Placebo | 45 (16.9%) [12 drug-related, 33 unrelated] | 18 (6.7%) | 21 (7.9%) | Protocol violation: 14 (5.2%) | Other: 12 (4.5%) | Reasons pooled across arms, from CONSORT diagram |
| Guttman-Yassky et al., 2020 | Upadacitinib, Placebo | 8 (4.8%) | 22 (13.1%) | 4 (2.4%) | 0 (0.0%) | Progressive disease: 2 (1.2%). LTFU: 2 (1.2%). Other: 2 (1.2%) | Reasons pooled across arms, from CONSORT diagram, totals reported |
| BREEZE-AD7 | Baricitinib, Placebo | 4 (1.2%) | 5 (1.5%) | 9 (2.7%) | 0 (0.0%) | 2 (0.6%) [Placebo group:1 patient failed screening and was randomized in error, and 1 patient was noncompliant with study visits] | Reasons reported by arm, totals calculated across all groups, From CONSORT diagram |
| Thaçi et al., 2016 | Dupilumab (300mg QW), Placebo | 4 (3.2%) | 14 (3.7%) | 4 (1.1%) | 1 (0.3%) | 6 (1.6%) | Reasons pooled across arms, categories may overlap |
| Guttman-Yassky et al., 2020 | Lebrikizumab, Placebo | 9 (3.2%) | NR | 41 (14.6%) | Protocol deviation: 2 (0.7%) | LTFU: 21 (7.5%). Pregnancy: 2 (0.7%). Physician decision: 3 (1.1%). Other: 2 (0.7%) | From CONSORT diagram, percentages were calculated against N=280 |
| Ruzicka et al., 2017 | Nemolizumab, Placebo | 10 (4.7%) | 7 (3.3%) | 15 (7.1%) | NR | LTFU: 2 (0.9%) | Reasons pooled across arms - arm-specific discontinuation rates reported, but reasons not stratified by dose. From CONSORT diagram, four EMA-relevant Q4W arms only (excludes 2.0mg/kg Q8W arm) |
| LIBERTY AD CHRONOS* | Dupilumab, Placebo | 35 (4.7%) | 30 (4.1%) | Included in Other | Protocol violation: 31 (4.2%) | 47 (6.4%) - includes withdrawal by participant, LTFU, and physician's decision, not separately reported at this level | Reasons reported pooled across arms. non-AE reasons dominated by lack of efficacy and disease worsening, particularly in placebo. From CONSORT diagram, week-52 layer. |
| BREEZE-AD1 | Baricitinib, Placebo | 2 (0.3%) | 18 (36.7%) | 24 (3.8%) | NR | LTFU: 1 (2.0%). Other: 4 (8.2%) | Reasons pooled across arms; Week 16 discontinuation counts reported, From CONSORT diagram |
| BREEZE-AD2 | Baricitinib, Placebo | 8 (1.3%) | 22 (3.6%) | 12 (2.0%) | NR | LTFU: 1 (0.2%). Other: 2 (0.3%) | Reasons pooled across treatment arms; arm-specific discontinuation rates reported separately, From CONSORT diagram |
| Heads Up trial | Upadacitinib, Dupilumab | 5 (0.7%) | NR | 12 (1.8%) | COVID-19 logistics: 1 (0.1%) | LTFU: 4 (0.6%). COVID-19 logistics: 1 (0.1%). Other: 5 (0.7%) | Reasons reported by treatment arm and pooled across arms. From CONSORT diagram, through week 16 only |
| JADE DARE | Abrocitinib, Dupilumab | 19 (2.6%) | 2 (0.3%) | 22 (3.0%) | Protocol deviation: 7 (1.0%). Medication error (no AE): 1 (0.1%) | LTFU: 6 (0.8%). Died: 2 (0.3%). Other: 7 (1.0%) | Reasons reported by treatment arm and pooled; percentages calculated using total randomized population (n=727). From CONSORT diagram. |
| Silverberg 2020 JACI | Nemolizumab, Placebo | 8 (3.5%) | 2 (0.9%) | 29 (12.8%) | Protocol violation: 1 (0.4%) | LTFU: 4 (1.8%) | Reasons largely pooled across arms AE counts reported by dose, other reasons not stratified |
| DUPISTAD | Dupilumab, Placebo | 4 (2.1%) | 1 (0.5%) | 2 (1.1%) | NR | NR | Reasons reported by arm for AE, lack of efficacy, and withdrawal. Small number of discontinuations overall, Patient disposition |
| Guttman-Yassky et al., 2019 | Baricitinib, Placebo | 11 (8.9%) | 13 (10.5%) | 7 (5.6%) | Protocol violation: 3 (2.4%) | LTFU: 3 (2.4%). Physician decision: 1 (0.8%). Other: 1 (0.8%) | Reasons pooled across arms. Percentages calculated using total study population (N=124) |
| SOLO 1 | Dupilumab, Placebo | 22 (3.3%) | 18 (2.7%) | Included in Other | Protocol violation: 3 (0.4%) | 39 (5.8%) - includes withdrawal, LTFU, physician decision, | Reasons pooled across treatment arms. Percentages calculated using total randomized population (N=671), excludes the deeper "did not complete study" (extension follow-up) breakdown, which is out of scope |
| SOLO 2 | Dupilumab, Placebo | 20 (2.8%) | 21 (3.0%) | Included in Other | Protocol violation: 11 (1.6%) | 25 (3.5%) | Reasons pooled across all treatment arms; percentages calculated using total randomized population (N=708) |
| ECZTRA 1 | Tralokinumab, Placebo | 28 (3.5%) | NR | Included in "other" reasons | Included in "other" reasons | 41 (5.1%) (Tralokinumab 31 [5.1%], Placebo 10 [5.0%]). Combined withdrawal, lack of efficacy, protocol deviation, and LTFU, not separately reported | Reasons reported both pooled and by treatment arm: lack-of-efficacy-specific discontinuation not separately reported |
| ECZTRA 2 | Tralokinumab, Placebo | 12 (1.5%) | NR | Included in "other" reasons | Included in "other" reasons | 43 (5.4%) (Tralokinumab 24 [4.0%], Placebo 19 [9.5%]). Combined withdrawal, lack of efficacy, protocol deviation, and LTFU, not separately reported | Reasons reported both pooled and by treatment arm: lack-of-efficacy-specific discontinuation not separately reported |
| ECZTRA 7 | Tralokinumab, Placebo | 4 (1.4%) | NR | NR | NR | 26 (9.4%) (Tralokinumab 12 [8.6%], Placebo 14 [10.2%]): combined withdrawal, lack of efficacy, protocol deviation, and LTFU, not separately reported | Non-AE reasons were not further stratified and likely include lack of efficacy, patient choice, and protocol deviations. Total discontinued minus AE-related, by arm. |
| LIBERTY AD CAFÉ | Dupilumab, Placebo | 4 (1.2%) | 3 (0.9%) | 1 (0.3%) | 2 (0.6%) | Included physician decision and post-treatment discontinuation | Reasons reflect most proximate cause. Some discontinuations occurred after treatment completion but before study end. From CONSORT diagram |
| BREEZE-AD4 | Baricitinib, Placebo | 17 (3.7%) | 110 (23.8%) | 21 (4.5%) | 2 (0.4%) | LTFU: 2 (0.4%). Other: 8 (1.7%) | Reasons reported across arms; lack of efficacy was the predominant cause of discontinuation, particularly in the placebo arm |

*Reflects 52-week data. This trial did not report 16-week discontinuation reasons on the CONSORT diagram.

**Supplementary Table S15.** Domain-level-risk-of-bias assessment (Cochrane RoB 2) for all included randomized controlled trials.

| **Study ID** | **D1** | **D2** | **D3** | **D4** | **D5** | **Overall** |
| --- | --- | --- | --- | --- | --- | --- |
| JADE COMPARE | Low | Low | Some concerns | Low | Low | Some concerns |
| Zhao et al., 2022 | Low | Low | Some concerns | Low | Low | Some concerns |
| TREBLE | Low | Low | Some concerns | Low | Low | Some concerns |
| ECZTRA 3 | Low | Low | Some concerns | Low | Low | Some concerns |
| Gooderham et al., 2019 | Low | Low | Some concerns | Low | Low | Some concerns |
| Guttman-Yassky et al., 2020 (upadacitinib) | Low | Low | Some concerns | Low | Low | Some concerns |
| BREEZE-AD7 | Low | Low | Low | Low | Low | Low |
| Thaçi et al., 2016 | Low | Low | Some concerns | Low | Low | Some concerns |
| Guttman-Yassky et al., 2020 (lebrikizumab) | Low | Low | Some concerns | Low | Low | Some concerns |
| Ruzicka et al., 2017 | Low | Low | Some concerns | Low | Low | Some concerns |
| LIBERTY AD CHRONOS* | Low | Low | Some concerns | Low | Low | Some concerns |
| BREEZE-AD1 | Low | Low | Some concerns | Low | Low | Some concerns |
| BREEZE-AD2 | Low | Low | Some concerns | Low | Low | Some concerns |
| Heads Up trial | Low | Low | Low | Low | Low | Low |
| JADE DARE | Low | Low | Low | Low | Low | Low |
| Silverberg 2020 JACI | Low | Low | Some concerns | Low | Low | Some concerns |
| DUPISTAD | Low | Low | Low | Low | Low | Low |
| Guttman-Yassky et al., 2019 | Low | Low | Some concerns | Low | Low | Some concerns |
| SOLO 1 | Low | Low | Some concerns | Low | Low | Some concerns |
| SOLO 2 | Low | Low | Some concerns | Low | Low | Some concerns |
| ECZTRA 1 | Low | Low | Low | Low | Low | Low |
| ECZTRA 2 | Low | Low | Some concerns | Low | Low | Some concerns |
| ECZTRA 7 | Low | Low | Some concerns | Low | Low | Some concerns |
| LIBERTY AD CAFÉ | Low | Low | Some concerns | Low | Low | Some concerns |
| BREEZE-AD4 | Low | Low | Some concerns | Low | Low | Some concerns |

# 3 References

1. European Medicines Agency. European public assessment reports (EPAR) [Internet]. Amsterdam: EMA; [cited 2026 Sep 24]. Available from: <https://www.ema.europa.eu/en/medicines>
2. Wan X, Wang W, Liu J, Tong T. Estimating the sample mean and standard deviation from the sample size, median, range and/or interquartile range. BMC Med Res Methodol. 2014;14:135. doi:10.1186/1471-2288-14-135
